# Design and Characterization of DX‐Tile DNA Nanostar‐Based Hydrogels

**DOI:** 10.1002/advs.202514506

**Published:** 2026-02-10

**Authors:** Dylan V. Scarton, Alessandra B. Coogan, Peter M. Touma, Eray O. Tulun, Katie A. Harrison, Jack Buchen, Richard C. Steiner, Christopher R. Fellin, Hunter G. Mason, Chih‐Hsiang Hu, Sally Farag, Xiaoning Yuan, Shailly Jariwala, Remi Veneziano

**Affiliations:** ^1^ Interdisciplinary Program in Neuroscience College of Science George Mason University Fairfax Virginia USA; ^2^ Institute for Advanced Biomedical Research George Mason University Manassas Virginia USA; ^3^ Department of Bioengineering College of Engineering and Computing George Mason University Fairfax Virginia USA; ^4^ Henry Jackson Foundation for the Advancement of Military Medicine, Inc. Bethesda Maryland USA; ^5^ Department of Physical Medicine and Rehabilitation Center for Rehabilitation Sciences Research Uniformed Services University of the Health Sciences Bethesda Maryland USA; ^6^ School of System Biology College of Science George Mason University Manassas Virginia USA; ^7^ Center for Infectious Diseases George Mason University Manassas Virginia USA

**Keywords:** bioprinting, DNA hydrogels, DNA nanotechnology, double‐crossover (DX)‐tile

## Abstract

Pure deoxyribonucleic acid (DNA) hydrogels synthesized via the hybridization of multi‐arm DNA tiles (DNA nanostars) are uniquely programmable and functionalizable biomaterials, suitable for applications ranging from biosensing to cell‐free protein production and soft tissue engineering. However, the full potential offered by DNA molecules in terms of design flexibility and functionalization has not yet been leveraged for pure DNA hydrogels, thus reducing their versatility and broader use. In this study, we introduce multi‐arm double‐crossover (DX)‐tile motifs, often used in wireframe DNA nanoparticles assembly, to enable greater control over the hydrogel's mechanical properties and facilitate functionalization. Specifically, we demonstrate that modifying structural design parameters, such as the arm geometry, length, valency, and linker design, allows for fine control of the elastic modulus and viscoelastic properties of the hydrogels. We also show that their functionalization can be performed without compromising the hydrogels' physical properties and exhibit enhanced mechanical strength and tunable properties, compared to simple duplex‐based DNA hydrogels. Furthermore, these DNA hydrogels demonstrated printability and scalability, which pave the way toward the development of novel formulations and bioinks for the rational design of soft tissue engineering scaffolds and broaden the use of DNA hydrogels for other biomedical applications.

## Graphical abstract

In this study, the authors demonstrated that DNA DX‐tile‐based multi‐arm motifs can be used to assemble pure DNA hydrogels, offering greater design flexibility and enhanced control over their mechanical properties and functionalization capabilities compared to single duplex‐based DNA hydrogels. They also showed that these hydrogels are printable, supporting their scalability.

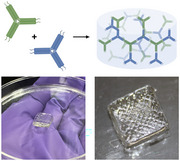

## 1 Introduction

Deoxyribonucleic acid (DNA) molecules have unique structural, mechanical, and biochemical attributes that can be leveraged to assemble biomaterials from the nano‐ to the macro‐scale with tunable physical properties and precise functionalization [1, 2, 3]. Notably, DNA molecules can be used to synthesize DNA‐based hydrogels and pure DNA hydrogels. DNA‐based hydrogels are generally composed of polymers such as polyethylene glycol diacrylate [4], ethylene diglycidyl ether [5, 6], or oxidized alginate [7] crosslinked with DNA duplexes or various stimuli‐responsive DNA motifs like G‐quadruplexes [8] and i‐motifs [9] to create dynamic 3D polymeric meshes [10]. These hybrid hydrogels have been successfully used as responsive biomaterials for 3D cell culture [11], tissue engineering [12], and soft robotics [13].

Pure DNA hydrogels, on the other hand, are exclusively comprised of nucleic acid molecules (e.g., folded 2D DNA motifs or long single strands of DNA produced by rolling circle amplification) that act as building blocks to form 3D polymeric meshes [14, 15, 16]. Among these hydrogels, multi‐arm DNA tile hydrogels, formed via the hybridization of 2D single duplex‐based DNA motifs bearing complementary overhangs, are one such type that have garnered great interest due to their superior programmability, addressability, and design flexibility in comparison with other pure DNA hydrogels [17, 18, 19]. These individual motifs, also known as DNA nanostars (DNAns), usually feature three or more arms that each comprise a single DNA duplex with lengths of about 20 nucleotides (nts) for a motif diameter of roughly seven nanometers (nm) [20]. Their mechanical and structural properties can be tuned by adjusting formulation parameters such as the initial motif concentration, as well as structural features including motif arm valency and the flexibility or length of the linker [21, 22, 23, 24, 25]. Due to the intrinsic mechanical properties of the DNA motifs, the limited number of crosslinks created, and the weak nature of hydrogen bonds, multi‐arm DNA hydrogels are relatively soft materials with elastic moduli that typically range from a few pascals (Pa) to more than two thousand Pa [16, 26, 27, 28]. As for the DNA‐based hydrogels, the dynamic nature of certain stimuli‐responsive DNA motifs (e.g., G‐quadruplexes, aptamers, and i‐motifs) can be easily exploited to enable reorganization of the 3D network at room temperature in response to various stimuli such as pH variation and biomolecules [16, 29, 30]. However, despite the tremendous progress that has been made in the field DNA nanotechnology over the past several decades, pure DNA hydrogels have not yet leveraged the complete potential and design flexibility inherent to DNA molecules. Indeed, their design mainly relies on relatively ‘simple’ DNA‐duplex‐based motifs, which often require highly concentrated motif solutions that can exceed 500 µm [18, 31]. This not only limits their scalability but also their functionalization, which further restricts them to a narrow range of applications where complex structures and functions are not critical, such as biosensing and drug delivery [32, 33, 34].

In this study, we leveraged design strategies conventionally employed for DNA nanoparticle synthesis to enhance the design flexibility of DNAns hydrogels and facilitate their complex functionalization for subsequent applications in tissue engineering and beyond. Specifically, we replaced the single‐duplex‐based arm in the DNA motifs with double‐crossover (DX)‐tile motifs, common in constructing wireframe DNA origami nanostructures [35], to create DX‐DNAns hydrogels. DX‐tile motifs offer several advantages, such as increased stiffness, more functionalization sites, and improved design flexibility [36, 37]. Our results demonstrated that DX‐tile‐based motifs can be used to assemble hydrogels with mechanical properties similar to those of other pure DNAns hydrogels, featuring a range of elastic moduli from ∼0.1 kPa to more than 1.2 kPa, while using drastically reduced motif concentration to as low as 25 µm. Importantly, our design strategy enabled the tunability of these mechanical properties, as well as ease of functionalization, without significantly altering the formulation of the hydrogels and thus compromising their structural integrity. Moreover, we demonstrated that these DX‐DNAns hydrogels possess viscoelastic properties conducive to printability. Altogether, our results highlight the potential of DX‐DNAns hydrogels to serve as a ‘smart’ soft biomaterial platform.

## 2 Results and Discussion

### 2.1 Design and Formation of DX‐Tile DNAns Hydrogels

**FIGURE 1:**
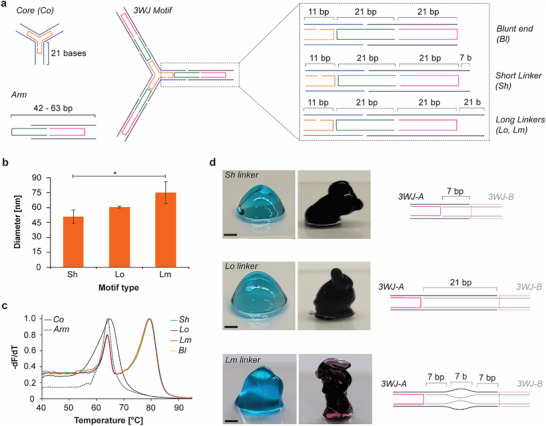
DX‐tile‐based DNA motif folding and DX‐DNAns hydrogel formation for 3WJ constructs. (a) Initial design of the DX‐DNAns constructs, showing the core and arm elements (*left*), and the different arm linkers (*right*). (b) Average diameter of 3WJ‐motifs with different linkers measured by dynamic light scattering. Error bars represent the standard deviation of the mean (*n* = 3 independent samples/group). *p*‐values were calculated by student *t*‐test (^*^: *p* <0.05) and presented in the Table S2. (c) Melting curves of motif constituents (*n* = 4 independent samples/group). (d) Representative array images of hydrogels assembled with motifs bearing different linker types and at different motif concentrations (60 µm, blue; 90 µm, black; scale bars: 1 mm).

To explore the influence of DX‐tile‐based DNA motifs on the physical properties of DNA hydrogels and facilitate comparison with previously published DNAns hydrogels, we first developed multiple variants of a three‐way junction (3WJ), comprised of a central core and three identical arms (Figure 1a; Figure S1a). Each 3WJ structure is formed using the tile assembly method to ensure proper folding of the motifs via the thermal annealing of multiple short oligonucleotide strands mixed at various molar ratios (Table S1). This synthesis strategy increased cost‐efficiency by enabling the reuse of the different oligonucleotides to explore various arm geometry combinations. To enable hybridization of multiple motifs, each 3WJ arm was modified with two short single‐stranded overhang linkers (one linker per duplex) that paired with complementary sequences on another 3WJ motif (Figure 1a; Figure S2 and Table S1). To evaluate the effect of linker length on hydrogel strength, we designed a short 7‐base overhang (Sh), to provide sufficient hybridization for gel formation [18, 22], as well as a long (Lo) 21‐base overhang, as it has been shown that longer linkers usually produce stronger hydrogels [18, 31]. We also designed a variant of the Lo linker with a non‐hybridizing middle region of 7‐base called the mismatch linker (Lm) (Figure 1a) to assess if flexibility and unhybridized regions in the linker decrease the mechanical strength of the DX‐tile DNA hydrogels, as previously shown [18]. We also designed a blunt end (Bl) version of the 3WJ that did not bear any ssDNA linkers (Figure 1a) to assess the role of base stacking in gel formation, as typically observed for DNA nanoparticles [38]. For all the 3WJ constructs, the arm‐to‐arm length without the linker is estimated at ∼31.6 nm with arm angles of ∼118.2° (Figure S3), while the same distance is approximated at ∼37.2 and ∼47.1 nm when including the Sh and Lo/Lm single‐stranded overhangs, respectively. After validating the sequences with NCBI BLAST [39] to avoid mispairings, the different parts of the 3WJ and the entire structures were slowly annealed, and their folding was assessed via agarose (AGE) and native polyacrylamide gel electrophoresis (PAGE). The gel images presented in Figure S4a–c demonstrate the assembly of monodispersed 3WJ core motif, arms, and of the three complete structures (with the Sh, Lo, and Lm linkers). However, it is important to note the presence of extra bands in the gels that may indicate the presence of some byproducts that represent either unincorporated oligonucleotides or incomplete structures for the low molecular weight bands, or multimers of motifs for the higher molecular weight bands that are often observed with multi arm tiles assembly [40]. The electrophoresis results are confirmed by atomic force microscopy (AFM) (Figure S4d). To further characterize the assembly of the different core and arm parts of the structures, we used a fluorescence resonance energy transfer (FRET)‐based experiment wherein a strand was modified with a Cy3 dye and another strand with a quencher. Moving the location of the Cy3 dye and quencher allowed us to validate the correct incorporation of all strands and the stepwise formation of the 3WJ motifs, with the arm flexibility affecting the FRET signal (Figure S5). Next, dynamic light scattering (DLS) was used to measure the hydrodynamic diameters of each motif. The average diameters obtained were 51.0 ± 6.7 nm, 60.6 ± 1.0 nm, and 75.1 ± 11.0 nm for 3WJ‐Sh, Lo, and Lm, respectively (Figure 1b). As expected, motifs with longer linkers (Lo and Lm) exhibited larger hydrodynamic diameters compared to the short linker. The deviations from the theoretical diameters are likely due to the flexible conformation of the linkers, the presence of dimerized motifs, and the limited accuracy of DLS in measuring the hydrodynamic diameter of non‐spherical and flexible DNA‐based assemblies [41].

We then determined the melting temperatures (Tm) of each motif to check for potential stability issues at physiological temperatures (Figure 1c). Our results show that the core and the arm have similar melting temperatures at 65°C and 64°C, respectively, which is typical for DX‐tile‐based assemblies [35]. For the fully formed motifs, two peaks are clearly visible at 64°C and 80°C due to the enhanced thermal stability induced by the cooperative assembly of the core and arms, as previously observed for similar DX‐tile geometries [35], which confirmed the formation of the entire structure. Altogether, these characterization results confirm proper assembly of all the motifs designed and assembled.

We then tested hydrogel formation by mixing complementary motifs at equimolar concentration ranging from 15 to 90 µm (Figure 1d). At 15 µm, none of the solutions containing complementary motifs appeared to form hydrogels. All formulations tested behaved like liquids spreading on the flat glass surface and flowed when the glass surface was tilted (Figure S6). At 30 µm, all tested motifs appeared more viscous than at 15 µm, which could indicate of the formation of a weak hydrogel (Figures S6 and S7). At higher concentrations, hydrogels clearly formed for all motifs, as shown in Figure 1d for hydrogels assembled with the Sh, Lo, and Lm linkers at concentrations of 60 µm (*blue*) and 90 µm (*black*).

The 90 µm samples consistently appeared to hold their shape better than the hydrogels formed at 60 µm across the three linker types tested, indicating higher mechanical strength. Although these results could be attributed to an increased concentration of the DNA motifs, mixing folded motifs at 60 µm with non‐complementary overhangs did not yield hydrogel formation, as seen in Figure S8. Interestingly, on the other hand, single Bl motif mixtures formed a hydrogel directly after folding of the individual motifs, as opposed to the other motif types that required proper mixing of the complementary motifs, likely due to blunt‐end stacking (Figure S9), as previously observed for DNA nanoparticles assembly [42, 43].

**FIGURE 2:**
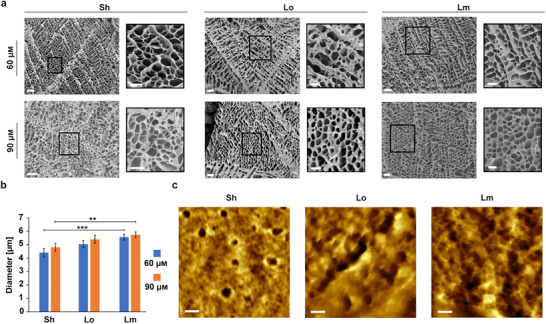
Structural characterization of the dried 3WJ hydrogels. (a) SEM images of the dried hydrogels (scale bar: 20 µm) with emphasis on the micro‐pore morphology (scale bar: 10 µm) (b) Micro‐pore size comparison across the various dried 3WJ hydrogels assembled with different linkers. Error bars represent the standard error of the mean (*n* = 50 independent samples/group). *p*‐values were calculated by student *t*‐test ^(**^: *p*< 0.01, ^***^: *p* <0.001) and presented in Table S4. (c) Nano‐pore morphology of the dried hydrogels observed by AFM (scale bar: 50 nm).

We used scanning electron microscopy (SEM) to characterize the hydrogels’ microstructure in a lyophilized state. As expected from visual observations, the 30 µm hydrogels exhibited a very loose fiber‐like mesh, likely due to the low concentration of motifs used, reducing its crosslinking ability (Figures S10 and S11). In contrast, the 60 and 90 µm hydrogels formed regular and uniform microporous mesh networks with smooth, round pores that are typically observed in other polymeric and pure dried DNA hydrogels [44, 45, 46] (Figure 2a; Figure S10b,c). To determine if the linker type could influence the porosity and network formation, we systematically measured the pore sizes for each hydrogel (Figure S12), which ranged from ∼4 to 6 µm in diameter (Figure 2b; Figure S13 and Table S3) across all three linker types. While the concentration ranges tested did not appear to significantly affect the pore size for any of the motifs tested, we noted a small increase of the pore diameters from Sh to Lm (Figure 2b). Of note, hydrogels formed with the 3WJ‐Bl motifs displayed similar pore sizes (Table S3). These results suggest that the micropore sizes in dried hydrogels were only marginally affected by the linker types within the range of motif concentrations tested, however based on our results it is impossible to conclude on the presence or the parameters of these micropores in fully hydrated samples. Furthermore, AFM imaging of the dried hydrogels (Figure 2c; Figure S14) revealed the presence of round and regular nano‐pores with smooth edges created from the nanoscale assembly of the motifs with similar diameters for 3WJ‐Sh (36.4 nm ± 7.9 nm), 3WJ‐Lo (35.0 nm ± 8.7 nm), and 3WJ‐Lm (41.9 nm ± 11.0 nm). These nanopores appear slightly larger than the nanopores previously observed with other DNA hydrogels using Y motifs, which might result from using longer arm lengths in our motifs [45, 47, 48]. Similarly to the micropores observed with SEM, it is important to note that because the samples were dried for the AFM measurements, the pore sizes might be different for the fully hydrated samples. Taken together, these results demonstrate that DX‐tile motifs can be used to form hydrogels. All tested linkers formed hydrogels at concentrations between 30 and 60 µm, including the base‐stacking crosslinks of the Bl arm terminus that formed with similar structural properties.

### 2.2 Nano‐Structural Features and Motif Concentration Alter Bulk Mechanical Properties

Next, we investigated the effects of the design elements for the DX‐tile nanostructure on the bulk viscoelasticity and mechanical strength of the hydrogels via rheological testing [49]. To facilitate comparison with the single duplex motifs typically used for DNAns hydrogels, we designed three‐armed single duplex motifs (Y motifs) with 7‐ (Y7) and 21‐ (Y21) base overhang linkers (Table S5 and Figure S15). The proper folding and monodispersity of the two Y motifs were confirmed by agarose gel electrophoresis (Figure S16a). Hydrogel formation was confirmed both visually (Figure S16b) and by SEM (Figure S16c) across motif concentrations ranging from 37.5 to 225 µm for Y7 and 30 to 180 µm for Y21. These ranges were selected to approximate some of the total DNA concentrations used in the DX‐DNAns hydrogels and to investigate how motif concentration, as opposed to total DNA concentration, influences hydrogel formation (Table S6).

**FIGURE 3:**
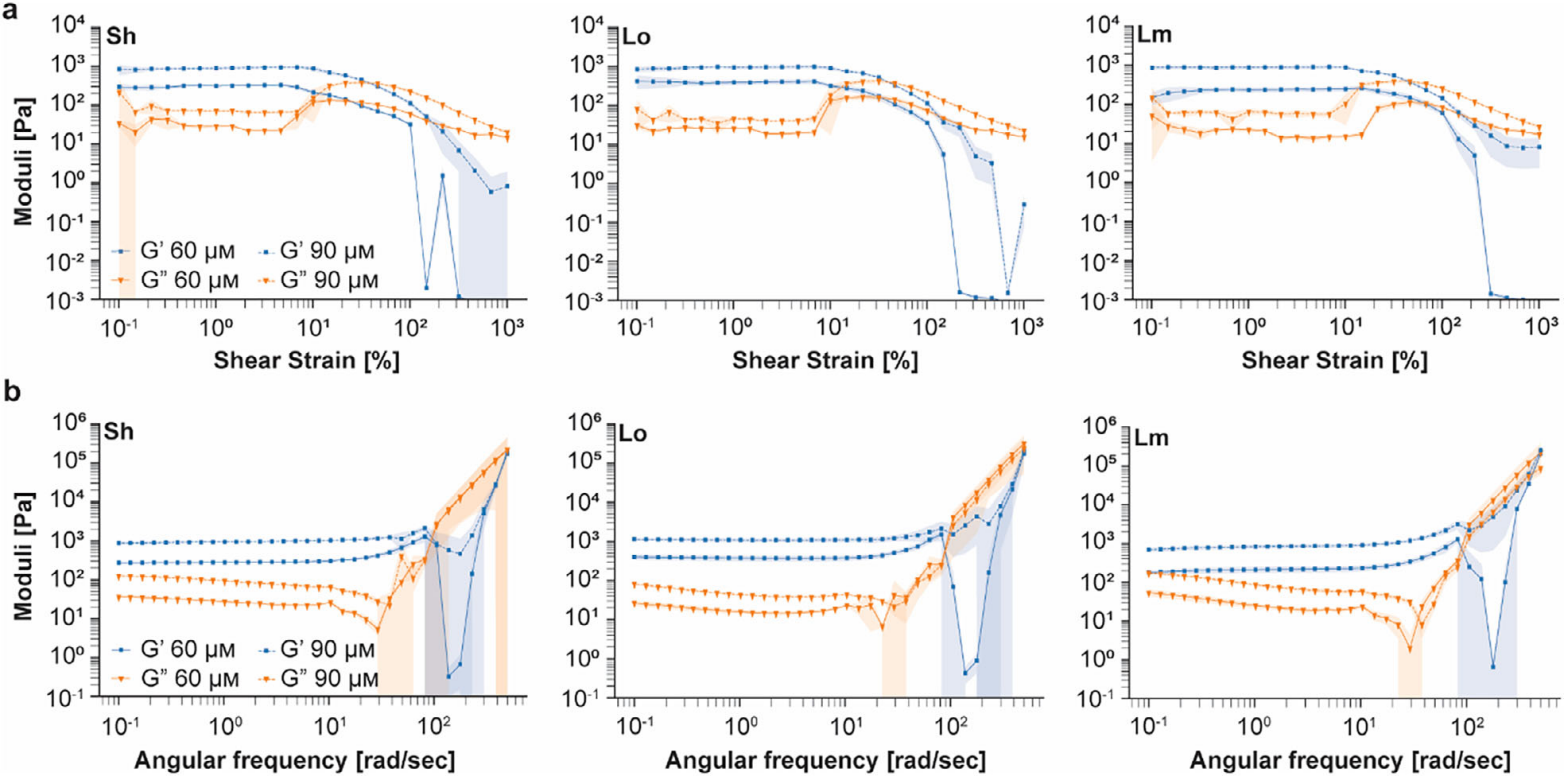
Rheological characterization profiles of the 3WJ constructs. (a) Amplitude sweeps of the hydrogels assembled at 60 and 90 µm of motifs with three different linkers. Error shadings represent the standard deviation of the mean (*n* = 3 independent samples/group). (b) Frequency sweeps of the hydrogels assembled at 60 and 90 µm of motifs with three different linkers. Error shadings represent the standard deviation of the mean (*n* = 3 independent samples/group).

We first performed amplitude sweeps (Figure 3a) to establish the linear viscoelastic range (LVER) and define the extent to which the hydrogel can be deformed under controlled oscillatory strain before yielding. The storage modulus (G′) represents the elastic, solid‐like behavior of the material, while the loss modulus (G″) represents its viscous, liquid‐like behavior. The experimentally‐determined modulus crossover or flow point is the strain at which the material transitions from dominant solid‐like behavior (G′ > G″) to dominant liquid‐like behavior (G″ > G′). The DX‐DNAns hydrogels folded at 60 and 90 µm with the Lm linker exhibited the highest modulus crossover points, while those with the Sh and Lo linkers had the lowest crossover points (Table 1). These results confirmed that using a flexible linker, namely the Lm linker, allowed for more elasticity, while using fully hybridized and more rigid linkers (Sh and Lo), reduced the overall flexibility of the hydrogel due to the more fully constrained and crosslinked networks, as observed for other pure DNA hydrogels in literature [50]. Overall, increasing the motif concentration differentially affects the LVER crossover point of the hydrogels based on the linker type. It is worth noting that this result is consistent with ranges observed for single‐duplex DNA hydrogels between ∼10%–80% [51] and similar DNA hydrogels between ∼6%–50% [50], as determined by material concentration and therefore hydrogel stiffness [50]. As a control, we performed amplitude sweeps on solutions of the single 3WJ‐Sh motif at 60 µm that did not yield hydrogels (Figure S17a), as shown with single 3WJ‐Lm in Figure S8. As expected with non‐hybridized DNA motifs, we did not observe a clear LVER, as the moduli inverted at multiple points and the plot showed considerable variance consistent with a non‐gel material. The rheological profiles of the DX‐DNAns hydrogels formed at 30 µm (Figure S18a), while more viscous than the singular motif, possessed non‐definitive LVERs in comparison with the 60 and 90 µm hydrogels, which indicate that the hydrogels formed at low concentration of motifs are quite weak (Figure 3a). This behavior is likely due to its stretched fiber‐like organization and defects also observed by SEM (Figures S10a and S11).

**TABLE 1:** Measured storage moduli (G′) and LVER crossover point for the hydrogels assembled with 3WJ at various concentrations and with different linkers constructs. Standard deviation shown as ± (*n* = 3 independent samples/group). All P values are available in Table S7.

Linkers	Measurements	3WJ 30 µm	3WJ 60 µm	3WJ 90 µm
Sh	Storage modulus G′ [Pa]	6 ± 2	296 ± 6	1010 ± 17
Sh	LVER Crossover point [%]	N/A	25 ± 3	38 ± 2
Lo	Storage modulus G′ [Pa]	21 ± 19	369 ± 56	1070 ± 129
Lo	LVER Crossover point [%]	N/A	33 ± 2	40 ± 5
Lm	Storage modulus G′ [Pa]	2 ± 1	226 ± 40	895 ± 100
Lm	LVER Crossover point [%]	N/A	64 ±6	44 ± 1.4

No definitive LVER was observed for the Y‐motif hydrogels at lower motif concentrations of 60 and 75 µm for the Y21 and Y7, respectively (Figure S19a). When evaluated at higher motif concentrations of 120 and 150 µm for the Y21 and Y7, respectively, the flow points occurred at ∼83% strain for Y7 and ∼104% strain for Y21 (*P* = 0.084) (Figure S19b), suggesting both nanostructure and linker design influence the LVER [50]. The LVER for these Y‐motif‐based hydrogels was greater than those of the DX‐tile hydrogels at a matching motif concentration, which is expected because the DX‐tile motifs require more energy to dehybridize their two linkers.

Following those characterizations, we conducted frequency sweeps (Figure 3b) to capture the frequency‐dependent rheological properties of the hydrogels. We observed distinct rheological profiles emerging across all the formulations and conditions tested, compared to a standard measured at 1 hertz (Hz) (Table 1). For the 60 and 90 µm hydrogels, the Lo linker yield the highest G′, while those formed with the Lm linker had the lowest G′ (Table 1). This trend is the opposite of the amplitude sweep results, which suggests an inverse relationship of the LVER and the G′, as described in other work related to DNA hydrogels where greater rigidity of the DNA scaffold increased DNA hydrogel stiffness [50]. Furthermore, the formation of supramolecular DNA hydrogels is also dependent on the rigidity of the network [51]. As a control, we evaluated the solution of a single 3WJ‐Sh motif at 60 µm that did not yield a hydrogel (Figure S17b). The G′ magnitude for the 30 µm hydrogels was low for all linkers, likely due to its incomplete formation with the Lo linker having the highest G′ and Lm the lowest G′ (Figure S18b; Table 1). Thus, for all linkers tested, our results demonstrate a concentration‐dependent effect. These moduli are comparable in magnitude to other DNA hydrogels on the order of a few hundred Pa [52], but require much less material and motif concentration (∼0.2%–0.3% solid content) to match and even exceed properties of similar constructs [50].

For the Y‐motif‐based hydrogels, as noted for the amplitude sweeps, they did not appear to form well at the lower motif concentrations of 60 and 75 µm (Figure S19a), but formed at higher concentration with G′ recorded at 90 ± 10 and 49 ± 33 Pa for of Y7‐150 µm and Y21‐120 µm, respectively (Figure S19b). Unlike the DX‐based hydrogels, it has been proposed that the longer overhang for single‐duplex DNA hydrogels may exceed the optimal range for gel formation [27]. Additionally, these results confirm that with lower motif concentration, our DX‐DNAns hydrogels possess enhanced mechanical strength over the Y‐motif‐based hydrogels and other peer designs while offering greater tunability. Moreover, it also confirmed that the total DNA concentration (Table S6) is not the main factor affecting the mechanical properties of the DX‐tile DNA hydrogels but the nano‐structural features and the concentration of the motifs. These results are consistent with what is known about the critical influence of the DNA structure on its physical features, such as its persistence length (∼50 nm [53] or 150 bp) and the rigidity of the DX backbone, which is twice that of linear duplex DNA [25].

To determine the effect of temperature on the formation of the hydrogels, we compared the rheological properties of 3WJ‐Sh hydrogels measured immediately after formation and following a 12‐min temperature cycle consisting of heating to 55°C (to ensure melting of the linkers only) then cooling back to room temperature to induce reassembly (Figure S20, Tables S8 and S9). After one heat cycle and a direct measurement without a mixing step, the G′ of the 3WJ‐Sh hydrogels decreased by ∼25% for 60 µm and ∼22% for 90 µm (Table S8), demonstrating the stability and self‐healing capability of these hydrogels. Interestingly, we observed a more drastic effect of nearly 75% change with Y7‐based hydrogels shifting from 89.8 ± 10.0 Pa pre‐heat cycle to 23.4 ± 11.2 Pa post‐heat cycle (Figure S21). These differences in the reduction of G′ for 3WJ‐Sh and Y7 hydrogels might be explained by the higher thermal stability provided by the two linkers in the 3WJ, compared with the single linker for the Y7.

Given that the rigidity and other physical properties of DX‐tile‐based nanoconstructs are dependent on their structural design, including crossover density and arm length [54, 55, 56, 57], we tested 3WJ‐Sh constructs with different crossover densities (Table S1 and Figure S22a) and arm lengths (Figure S1b, Tables S10 and S11). For the crossover densities, the G′ was similar for hydrogels possessing one extra crossover in the middle of the arm (274 ± 45 Pa for 3WJ‐Sh‐1X vs. 296 ± 6 Pa for 3WJ‐Sh; Figure S22b). However, the G′ was extremely low for constructs with an extra double crossover (14 ± 4 Pa for 3WJ‐Sh‐2X; Figure S22c). This difference could be explained by the fine balance necessary between arm rigidity to increase stiffness and flexibility to permit hybridization with other motifs in a 3D mesh [25, 51, 58]. For the arm lengths, the short arm hydrogels composed of repeating sequences with either an outside (Figure S1b(i)) or inside (Figure S1b(ii)) crossover did not appear to form a hydrogel (Figure S23a,b). However, when using a non‐repeating sequence with an inside crossover that provides more flexibility to the arm (Figure S1b(iii)), the resultant hydrogel had the same crossover point as the long arm variant (Figure S1c) at 102% (Figure S23c,d). The arm length did affect the storage modulus of the hydrogels, as those formed with the non‐repeating, inside‐crossover variant had a lower modulus (49.2 ± 6.7 Pa; Figure S23c) than those formed with the longer arm variant (184 ± 73 Pa) (Figure S23d). These values were lower than the comparable regular arm analogue and could be explained by the overall flexibility of these two variantsin comparison with the regular arm.

### 2.3 Arm Valency in DX‐Tile Based DNA Hydrogels Alters Bulk Mechanical Properties

Next, we explored how the arm valency could impact the bulk material properties. Previous studies have shown that the valency of crosslinkers drastically influenced the physical properties of the hydrogels formed, likely due to its greater crosslinking density [59, 60]. We proceeded in the same fashion as with the 3WJ by first designing a six‐way junction (6WJ) central core (Co) that comprised the vertex of the full motif unit (Table S12 and Figure S1f) and was capable of displaying the same linker types (Figure S24a). Its arm‐to‐arm length was estimated at 20.7 nm and the measured arm angle at 64.5° (Figure S24b). We confirmed proper folding of the Co and full motifs with gel electrophoresis (Figure S24c,d) that showed some byproducts as observed for the 3WJ motifs, and AFM imaging of the individual 6WJ motifs (Figure S24e). Additionally, we determined the melting temperatures (Figure S24f) of the individual Co at ∼68°C, the arm at ∼67°C, and the fully folded motifs with peaks at ∼62°C, ∼68°C, and 80°C, as measured for the 3WJ motifs showing assembly of the full motifs.

**FIGURE 4:**
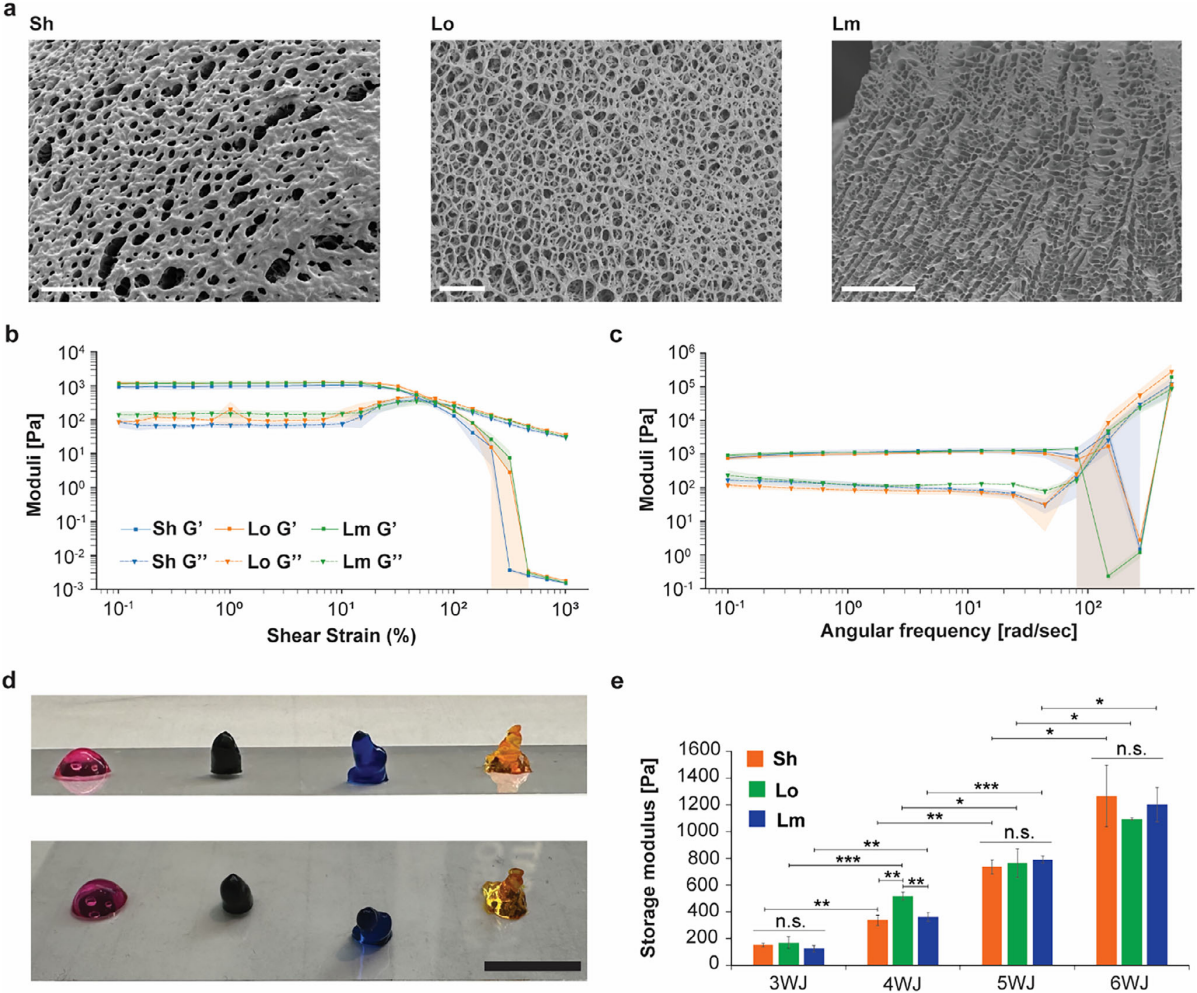
Arm valency in DX‐tile‐based DNA hydrogels alters bulk mechanical properties. (a) Representative SEM images of dried 6WJ hydrogels (scale bar: 50 µm). (b) Amplitude sweeps for all 6WJ hydrogels at 50 µm. Error shadings represent the standard deviation of the mean (*n* = 3 independent samples/group). (c) Frequency sweeps for all 6WJ hydrogels at 50 µm. Error shadings represent the standard deviation of the mean (*n* = 3 independent samples/group). (d) Hydrogel array for the 3‐, 4‐, 5‐, and 6WJ constructs at 50 µm (Scale bar: 1 cm). (e) Storage modulus for all hydrogels formed at 50 µm. Error bars represent standard deviation (*n* = 3 independent samples/group). All *p*‐values (displayed and non‐displayed on the graph) were calculated by student *t*‐test (not significant, n.s.; ^*^: *p* <0.05; ^**^: *p* <0.01; ^***^: *p* <0.001) and presented in Table S13 for clarity.

As with the 3WJ hydrogels, we tested the formation of the 6WJ hydrogels with the Sh and Lo linkers by mixing complementary motifs at equimolar concentration ranging from 7.5 to 45 µm (Figure S25). These concentrations were chosen to match the total DNA concentration to that of the 3WJ hydrogels since 60 and 90 µm motif concentrations were not possible using our working stock concentration for the 6WJ hydrogels given the additional number of strands required per motif (16 strands for 3WJ vs. 33 strands for 6WJ). The formation of hydrogels was not seen for the low concentrations (7.5 and 15 µm) of either motif tested but hydrogels were clearly formed for both motifs at 30 and 45 µm, with a clear concentration dependence on their apparent mechanical strenghts. SEM analysis of the dried hydrogels (Figure 4a; Figure S26a) further confirmed hydrogel formation across the different linkers, including the Bl linker, and different concentrations with clear and extensively uniform microporous networks (Figure S27). While the measured Y7 (225 µm) and 3WJ‐Lm (90 µm) pore sizes were similar (5.8 ± 1.5 µm vs. 5.7 ± 0.2 µm, respectively), the 6WJ‐Lm (50 µm) pore size was significantly less at 3.9 µm (*p* <0.001). This result suggests that the motif valency extent of crosslinking seem to positively affect the reduction in hydrogel pore size. Compared to the general 3WJ pore size of ∼5–6 µm, the 6WJ pore size of <4 µm presents another morphological feature that can be modulated along with mechanical properties.

We performed a series of amplitude sweeps (Figure 4b; Figure S26b) to ascertain the LVER for the three hybridizing DX‐tile motifs at two motif concentrations that were comparable to some of those tested for the 3WJ. For the 6WJ motif, the LVER crossover points were consistent across all three linkers and at both concentrations tested. As observed with the 3WJ hydrogels, the Sh and Lo linkers exhibited lower crossover points than the more flexible Lm linker (Table 2). However, the difference between the concentrations was not as pronounced as the 3WJ, suggesting the influence of the valency (3WJ vs. 6WJ) is greater than that provided by the motif concentration.

**TABLE 2:** Measured storage moduli (G′) and LVER crossover point for the hydrogels assembled with 4WJ, 5WJ, and 6WJ with different linkers. Standard deviation shown as ± (*n* = 3 independent samples/group). All *p*‐values are available in Table S13.

Motifs	Measeurements	3WJ 50 µm	4WJ 50 µm	5WJ 50 µm	6WJ 25 µm	6WJ 50 µm
Sh	Storage modulus G′ [Pa]	152 ± 13	337 ± 38	736 ± 53	119 ± 12	1266 ± 230
Sh	LVER crossover point [%]	26 ± 4	24 ± 0.2	25 ± 7	37 ± 6	58 ± 9
Lo	Storage modulus G′ [Pa]	171 ± 44	518 ± 29	764 ± 107	179 ± 46	1095 ± 8
Lo	LVER crossover point [%]	50 ± 12	31.9 ± 1.2	52 ± 5	41 ± 6	60 ± 8
Lm	Storage modulus G′ [Pa]	124 ± 24	363 ± 34	791 ± 26	282 ± 25	1201 ± 128
Lm	LVER crossover point [%]	68 ± 21	50 ± 2	51 ± 13	56 ± 9	71 ± 14

For the frequency sweeps (Figure 4c; Figure S26c), G′ increased in magnitude for all of the samples relative to their 3WJ counterparts. For the 25 µm hydrogels, those formed with the Lm linker had the highest G′ at 1 Hz, while those with the Sh linker had the lowest G′ and those with the Lo linker had a G′ in‐between the others. This pattern matched that of the amplitude sweep for both hydrogel concentrations (Lm > Lo > Sh). However, this trend significantly reversed for the 50 µm hydrogels, where those formed with the Sh linker had the highest storage modulus, while those with the Lo linker had the lowest storage modulus, and those with the Lm linker fell in‐between. This concentration‐dependent shift to Sh > Lm > Lo may result from the increased crosslinking creating a cooperative effect, as these higher moduli were all similar (*p* >0.3), and from the shorter overhang creating greater rigidity. As similar to the 3WJ samples, we performed a heat step and observed a more significant decrease in the storage moduli for the 25 µm and 50 µm to 29 ± 17 Pa (*P* = 0.004) and 347 ± 29 Pa (*P* = 0.005), respectively (Figure S28, Tables S8 and S9). The combination of a higher motif density and greater motif valency may have interfered with the crosslinking ability of complementary motifs to relocate one another after the additional heating step without extra mixing. A summary set of these storage moduli and are displayed in Table 2.

To enable more fine‐tuning of the storage modulus, we decided to test two other variants of the hydrogels, namely the four‐way junction (4WJ) and five‐way junction (5WJ) (Figure S1d,e, Tables S14 and S15). The addition of these designs would provide a deeper understanding of the effect of additional arms on the hydrogels’ physical and structural properties. As done for the 3WJ and 6WJ, we folded and confirmed the assembly of the motifs using electrophoresis and qPCR prior to forming the hydrogels. The results in Figure S29 demonstrate proper assembly of the core and full motifs for both 4WJ and 5WJ and Tm similar to those of the 3WJ and 6WJ. Interestingly, despite proper assembly of the full structures for both 4‐ and 5WJ motifs, we noted more byproducts for the 5‐ and the 6WJ motifs on the agarose and PAGE gels in comparison with the 3‐ and 4WJ motifs (Figures S4a–c, S24c,d, S29a,b,d,e), which can be due to the increasing complexity of these motifs. Rheology was conducted on these hydrogels at 50 µm to match the 3WJ and 6WJ concentrations and give insight into the effect that the number of arms has on the hydrogels’ overall strength. Our results show that with the sequential addition of arms, the storage modulus increases in a monotonic and significant way for all linkers types (Figure 4e and Table 2; Figure S30 and Table S13). The LVER crossover point appeared to be consistent across all motifs as well, with the use of the Sh linker generally resulting in a stiffer hydrogel, while the Lm results in hydrogels with a more elastic behavior.

To further assess the precise tunability of our system, we evaluated the effect of matching the valency ratios between the 3WJ and 6WJ motifs by combining them at 2:1 (3WJ:6WJ) and verified hydrogel formation by SEM (Figure S31a). We tested different motif ratios of 1:6, 1:1, and 6:1 to assess their impact on rheological properties. For the amplitude sweeps (Figure S31b), the 1:1 and 1:6 samples had similar LVER crossover points of 39% and 38%, respectively; however, for the 6:1 sample, it was much higher at 69%. Likewise, for the frequency sweeps (Figure S31c), the 6:1 sample had the highest G′ modulus at ∼1 Hz (416 ± 81 Pa), while a stepwise effect was observed for the 1:6 and 1:1 ratios from 270 ± 38 Pa to 347 ± 54 Pa, respectively. Interestingly, all combination ratios produced increased stiffness relative to their single motif counterpart. This outcome could be explained by the formation of a more branched network when using multiple motifs of different valencies, thus confirming the importance of the nanoscale organization of motifs. Moreover, adjusting valency ratios between motifs not only controls the mechanical properties of our hydrogels, but can also create tailored materials amenable to direct patterned functionalization by creating niches of controlled mechanical properties while assembling the hydrogel.

### 2.4 DX‐Tile DNA Hydrogels Demonstrate 3D Printability

**FIGURE 5:**
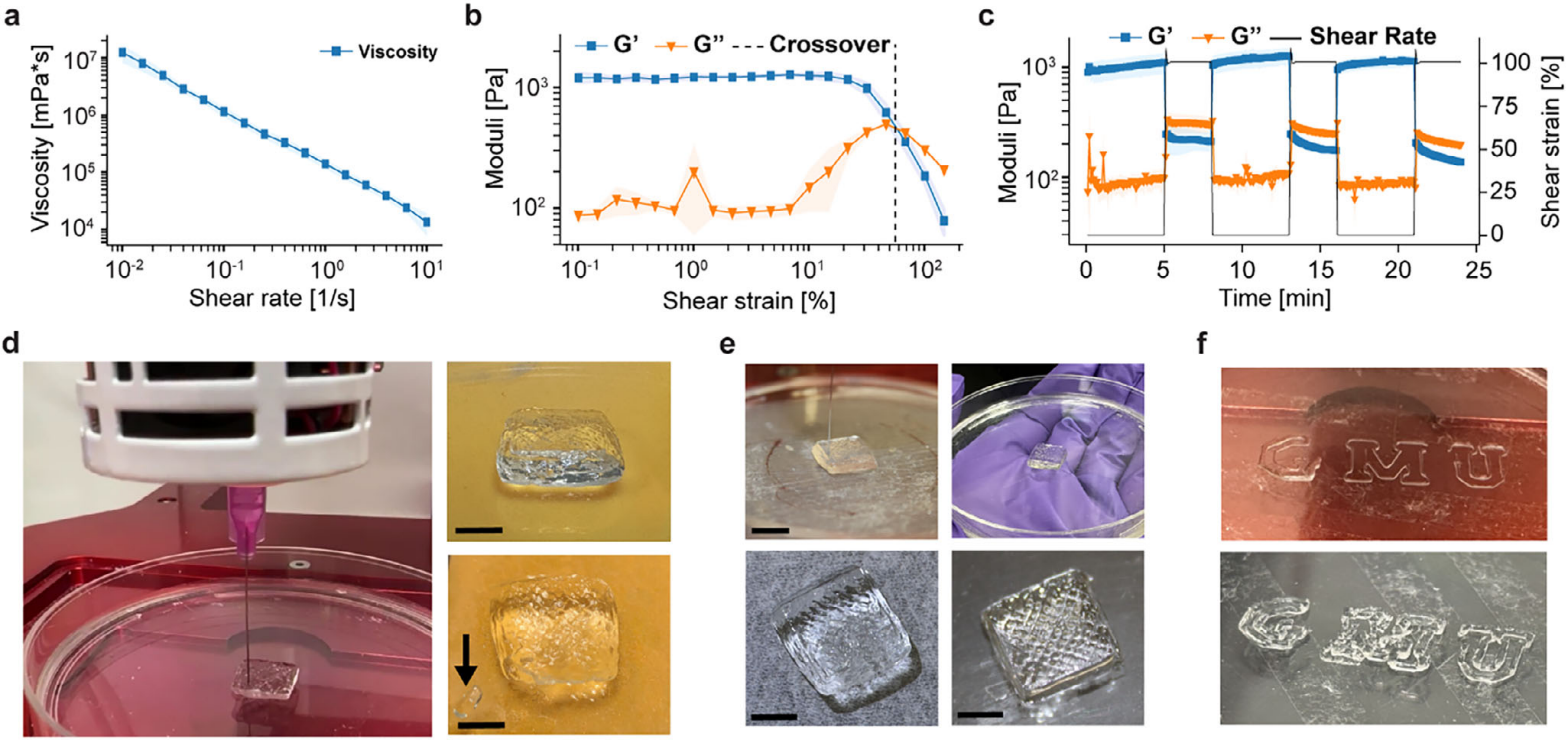
3D printing profile of 6WJ‐Lo constructs. (a) Viscosity curve indicated shear thinning behavior. Error shadings represent the standard deviation of the mean (*n* = 3 independent samples/group). (b) Amplitude sweep determined LVER. Error shadings represent the standard deviation of the mean (*n* = 3 independent samples/group). (c) Cyclic strain testing demonstrated recoverability. Error shadings represent the standard deviation of the mean (*n* = 3 independent samples/group). (d) First print with the Lm linker in process (*left*), after completion (*top right*), and after 12 h in 4°C with a piece removed (*arrow*) to show its integrity (*bottom right*) (scale bar: 1 cm). (e) First print with the Lo linker in process (*top left*), after completion (*bottom left*), and after collection and reprinting following a brief re‐annealing step at 55°C (*right top/bottom*) (scale bar: 1 cm). (f) GMU print with 6WJ‐Lo motif in process (*top*) and after completion (*bottom*).

The viability of DX‐DNAns hydrogels as extrudable bioink for 3D printing was also assessed. We first conducted rheological measurements to ensure that these new hydrogel formulations were suitable for extrusion 3D printing. We chose the 6WJ‐Lo DNA hydrogel (50 µm motif concentration) with a high elastic modulus (Table 2). We verified that the hydrogels were shear‐thinning (Figure 5a), confirmed the point at which their elastic behavior broke down and began to flow (Figure 5b), and studied if they recovered to their previous physical state (Figure 5c). Recoverability was of particular interest, as we had observed self‐healing behavior with this material following tears and cuts (Figure S32) as previously observed in literature for pure DNA hydrogels [61].

Once testing was complete for the basic material properties, we used an Allevi 3 extrusion printer to test the viability of 3D bioprinting the material. Various printing parameters were tested to identify a successful printing configuration: 25‐gauge (G) and 30 G syringes at 30, 35, and 40 pounds per square inch (PSI) were used to create prints at 1X, 2X, and 3X the inner diameter of the needle under 6, 9, and 12 mm/s print speeds (Figure S33). Following successful tests, an ideal combination of parameters was identified, and a 20 × 20 × 10 mm brick print shape was modeled using CAD and sliced for printing with 130 layers. The gel was printed with a 1‐inch, 30 G needle at 68 PSI and 6 mm/s speed (Figure 5d, left). After storage at 4°C for 12 h (Figure 5d, top right), we cut a piece of the hydrogel to confirm its integrity (Figure 5d, bottom right). Next, we printed the cube again and collected the material to immediately reprint the same design with no observable reduction in print quality, thus demonstrating the self‐healing properties intrinsic to these pure DNA hydrogels (Figure 5e) [62, 63]. Finally, to demonstrate the excellent print resolution and filament fidelity of our bioink, we printed the George Mason University (GMU) logo letters (Figure 5f).

### 2.5 DX‐DNAns Hydrogel Functionalization and Stability

**FIGURE 6:**
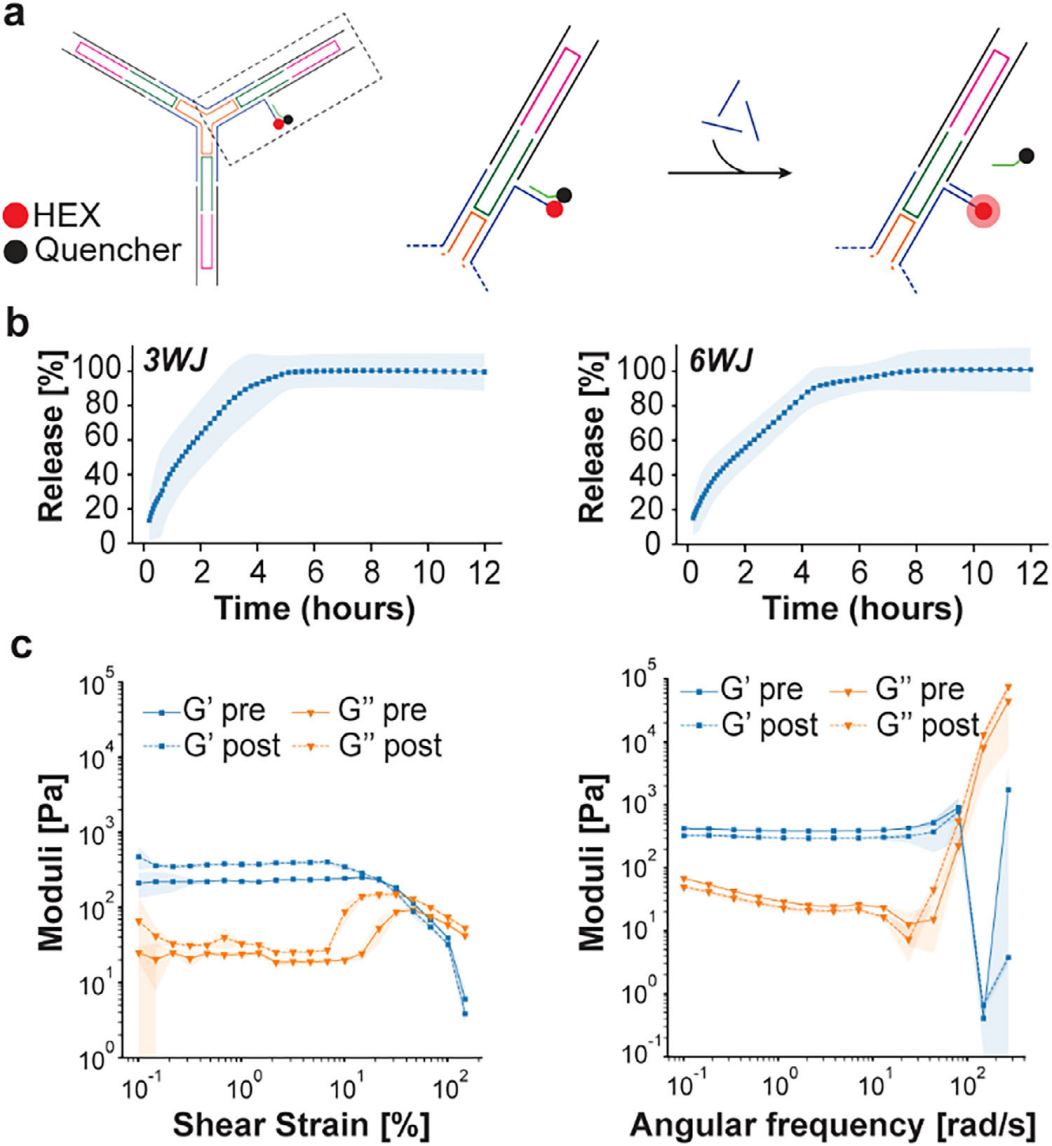
Hydrogel functionalization and stability. (a) Schematic diagram of motif modification for the strand displacement release system. (b) Release curve for 3WJ‐Lm (*left*) and 6WJ‐Lm (*right*) hydrogels at comparable concentrations of 60 and 30 µm, respectively. Error shadings represent the standard deviation of the mean (3WJ, *n* = 3, and 6WJ, *n* = 7 independent samples/group). (c) Amplitude (*left*) and frequency sweep (*right*) of functionalized 3WJ‐Lm hydrogels at 60 µm before and after cargo release with ssDNA triggers. Error shadings represent the standard deviation of the mean (*n* = 3 independent samples/group).

DNA motifs can be readily modified with a strand displacement reaction (SDR) system to enable the stimuli‐ and temporal‐controlled release of various cargos to create smart biomaterials [64, 65]. The release of cargo molecules directly attached to the motifs constituting the DNAns hydrogels can affect the mechanical properties and stability of these hydrogels [66, 67]. Here, we aim to demonstrate that our DX‐DNAns hydrogels can serve as a platform to design SDR systems capable of performing controlled release without affecting their mechanical properties or stability. Leveraging a previously published SDR circuit devised [68], we included a short overhang on one of the arm strands of our 3WJ and 6WJ DNA motif and conjugated it with a HEX fluorophore. This overhang was used to hybridize a cargo strand with an IowaBlack quencher (Figure 6a). This circuit was designed such that adding an excess of a trigger strand with greater affinity to the modified DNA motif than the quencher strand would displace the quencher over a period of time that could be quantified by an increase in fluorescence intensity.

With both constructs at comparable amounts of DNA content (3WJ‐Lm, 60 µm; 6WJ, 30 µm), we observed a similar cargo release between these two motif types, with only minor and non‐significant differences in their kinetic profiles (3WJ: 0.54 ± 0.25 h^−1^ vs. 6WJ: 0.44 ± 0.26 h^−1^; *p* = 0.69) and 50% release time (3WJ 1.29 ± 0.79 h vs. 6WJ 1.57 ± 1.41 h; *p* = 0.67) (Figure 6b; Table S16). This small difference in their release could be attributed to the smaller pore sizes and the denser 3D network within the 6WJ construct. To verify that functionalization did not disrupt the mechanical properties of the hydrogels, we performed rheology tests on the 3WJ hydrogels loaded with the SDR system before strand triggering and after release. As determined by amplitude (Figure 6c left) and frequency sweeps (Figure 6c right), the rheological profile remains largely unchanged after completely triggering the strand release in the hydrogel. The LVER crossover point shifted between pre‐ and post‐triggering from 32% to 60%, respectively, and G′ at ∼1 Hz changed from 391 ± 11.3 to 298 ± 15.8 Pa after total strand release. Of note, these values appear different than the values obtained for the unmodified hydrogels (Table 1), which can be explained by the functionalization of these motifs. Moreover, the ∼24% decrease in G′ is far less than previously reported for similar systems [51]. Additionally, to determine the long‐term stability of our hydrogels, we analyzed a 3WJ‐Sh hydrogel (60 µm) over the course of 5 days after assembly and noted that the the storage modulus at ∼1 Hz did not change between days 1 and 3 with values of 202 ± 33 Pa and 203 ± 32, respectively, and started to decrease slightly to reach 169 ± 31 Pa by day 5 (Figure S34). This small decrease (∼16%) might be due to spontaneous and dynamic reorganization of the hydrogel over time or the result of its slow degradation, but overall it demonstrated the stability of our hydrogels over the course of at least a few days.

## 3 Conclusion

DNA nanotechnology has allowed unprecedented control over the assembly of nanoscale DNA‐based motifs and particles and the precise patterning of various biological and non‐biological molecules to create highly functional nanoarchitectures. While significant progress has been made in the design and assembly of discrete DNA nanoparticles, the development of macroscale materials made purely from DNA has been limited to duplex‐based DNAns motifs and long hybridizing DNA. For instance, there has been very little exploration into more complex structures and design features, which can be used to tune the physical properties of these materials and facilitate their functionalization. Moreover, DNA nanostructures are limited to a few hundred nanometers in size, and remain relatively costly to produce, which fundamentally hinders the production of large‐scale materials [69, 70]. Furthermore, it is unclear to what extent these nanostructures can be linked to form macroscale materials and whether the structures or their crosslinks will remain stable.

In this study, we explored the impact of controlling the critical physical parameters of our DX‐DNAns hydrogels (i.e., linker type, arm design, and arm valency) to determine their effect on the bulk mechanical properties, while keeping the motif concentrations identical. Our results demonstrated that the nanoscale flexibility afforded by these DX‐tile motifs can produce programmable, self‐organizing macroscale hydrogels with highly tunable mechanical properties, including material stiffness ranging from a few Pa to more than 1.2 kPa. While this range is not higher than most of the previously designed DNAns hydrogels, it is important to note that these values are reached with a lower concentration of motifs and a lower concentration of DNA overall (Table S6), which reduces the overall cost of these biomaterials. It was also determined that the specific linker type used (Sh, Lo, or Lm) changed the elasticity of the hydrogel, while the storage modulus was primarily affected by the motif concentration and the number of arms in the motifs. Moreover, DX‐DNAns‐based hydrogels solve the limitations of previous iterations of pure DNA hydrogels, offering a unique medium to study soft robotics and polymeric materials with design control and efficient synthesis. In addition, given the similarity of the motif design with 3D DNA nanostructures, these hydrogels could enable large‐scale nanoscale organization and manipulation of various biomolecules for direct cell behavior and regulate lipid membranes, deliver drugs and targeted therapies, determine biophysical interactions, and analyze molecular structures [71, 72, 73]. From the nano‐ to macro‐scale, this unique strategy presents a desirable candidate for the rational design of hydrogels with prescribed mechanical properties that can be leveraged for biological applications as varied as studying viscoelastic influences on mechanobiology [34] to designing scaffolds for soft tissue engineering, such as adipose and nerve tissues [58].

## 4 Methods

### 4.1 Materials

All DNA oligonucleotides non‐modified and modified (i.e., Cy3, IowaBlack quencher, HEX) were purchased from IDT and directly used without further purification. All sequences used are listed in the Tables S1, S5, S10–S12, S14, S15. The folding buffer used to assemble the motifs is composed of Tris (40 mM), acetic acid (20 mM), ethylenediaminetetraacetic acid (2 mM) complemented with magnesium chloride (MgCl_2_) (12 mM) at pH 8.0 in nuclease‐free water (E476; VWR Life Science). For gel electrophoresis, high‐melt agarose (IB70070; IBI Scientific) was used between 1 and 1.5% with 1X Tris acetate EDTA buffer (pH 8.0) and pre‐stained with ethidium bromide (0.5 µg/mL) (15585‐011; Invitrogen) while samples were loaded with 1X Gel Loading Dye Purple (B7025S; New England BioLabs). Array samples were artificially colored with printer linker (X001OPYOXP; Aomya). Samples for the melting curves were stained with 1X SYBR Green I Nucleic Acid Gel Stain (50513; Lonza) and diluted in folding buffer. SEM samples were flash‐frozen in liquid nitrogen (Roberts Oxygen) and mounted with carbon tape (77825‐12; Electron Microscopy Sciences) on aluminum pedestals (RS‐MN‐10‐005112‐50; RaveScientific). Positive displacement pipettes were used for mixing and handling hydrogels.

### 4.2 Design of the DX‐Tile DNA Motifs

The DX‐tile‐based DNA motifs were designed with the Tiamat 2 program [74]. The strand sequences designed for each construction are available in the Tables S1, S5, S10–S12, S14, S15. To ensure that a designed strand sequence would only bind to its desired complementary sequence and avoid non‐specific binding, we used NCBI's Nucleotide BLAST [39].

### 4.3 oxDNA Modeling of the DNA Motifs and Visualization of the PDB Models

All DNA motifs created in Tiamat 2 were converted into PDB using the TacoxDNA tools suite [75]. The University of California San Francisco's Chimera 1.16 [76] was used to visualize and render the 3D molecular structure of the motif and measure the distances and angles.

### 4.4 Folding the DNA Motifs

The DNA motifs were folded in a one‐pot reaction with all strands added at equimolar concentration or at indicated molar ratios for strands used multiple times in a motif. The oligonucleotide mix was prepared in our 1X folding buffer and annealed from 90°C to 4°C overnight in a Biorad T100 thermocycler. Following folding, the nanostructures were stored at 4°C without further purification prior to characterization or gel formation. The concentration of the DNA motifs was measured using a Nanodrop One.

### 4.5 Gel Electrophoresis

To assess proper assembly of the motifs, folded DNA motifs were run on agarose gel pre‐stained with ethidium bromide at 100 V for 30 min or on polyacrylamide gel (6 or 12%) in Tris‐acetate EDTA buffer complemented with MgCl_2_ (12 mm) at 120 V for 90 to 120 min prior to be stained with the SYBR Safe gel stain, and imaged with an Azure c150 blue transilluminator. The Gel were analyzed in ImageJ [77].

### 4.6 Fluorescence Resonance Energy Transfer (FRET) Assay

Folded structures were validated via a FRET assay performed with a Cy3/IowaBlack dye quencher pair. Cy3 dye was excited at 555 nm and the maximum emission was monitored at 570 nm with a SpectraMax Gemini EM Microplate Reader from Molecular Devices. Samples were prepared at a volume of 40 µL into a 384‐well black flat transparent bottom plate.

### 4.7 DNA Hydrogel Formation

All hydrogels were assembled by mixing equal parts of one motif (A motif) with its complementary form (B motif) in 1X folding buffer. The only exception was the blunt‐end DNA motifs, which did not require a complementary moiety as they did not have linkers. The hydrogel concentrations reported refer to the total motif concentration.

### 4.8 Melting Curves

Melting curves were acquired using a qPCR instrument (The Q instrument from Quantabio). A total of 100 µL for each sample was pipetted evenly into a strip of four loading tubes (1.25 µL of SYBR Green I dye [1X final concentration], 12.5 µL of motif, 11.25 µL of folding buffer per tube). Light exposure was minimized to avoid bleaching of the dye. The tubes came preloaded from the supplier with silicone oil to prevent evaporation, condensation, and the need for a heated lid. The samples were melted from 40.0°C to 95.0°C at 0.05°C/s, for two cycles. Melting curves were extracted as Excel files, and the melting temperature was determined by taking the average of the fluorescent intensity for the quadruplicate samples. Data was then normalized against the peak value.

### 4.9 Atomic Force Microscopy Evaluation

AFM images were captured with a Bruker Dimension Icon + ScanAsyst. For imaging the DNA hydrogels, hydrogel samples (100 µL, 60 µm) were layered onto freshly cleaved mica surfaces of 15 mm diameter and a thickness in the range of 0.15–0.21 mm (Electron Microscopy Sciences) and left in a vacuum chamber overnight to dry. The samples were then imaged in ScanAsyst mode with a SCANASYST‐AIR‐HPI probe (Bruker). For DNA motif imaging, 20 µL of ∼1–5 nm folded DNA motifs in folding buffer were deposited onto freshly cleaved mica and incubated for 10 min, followed by several gentle folding buffer washes. The motifs were immediately imaged in a folding buffer supplemented with NiCl_2_ (10 mm), using ScanAsyst mode and a SNL‐10 probe (Bruker). All AFM images were processed using Gwyddion [78].

### 4.10 Dynamic Light Scattering Measurements

Hydrodynamic diameters of the folded 3WJ motifs were measured via DLS using a Malvern Zetasizer Nano ZS instrument. Samples (1 µm, 60 µL) were diluted with 1X folding buffer and loaded into disposable plastic cuvettes. Size measurements were taken with 173° backscatter.

### 4.11 Scanning Electron Microscopy Evaluation

SEM was used to confirm hydrogel formation with the JEOL JSM‐7200F instrument and PC‐SEM software. Formed hydrogels (40 µL) were directly cast into 3D‐printed resin rings (12‐mm diameter) that had been fixed onto brass pedestals with carbon tape and then freeze‐dried with at least 10 min of liquid nitrogen and 24 h of lyophilization. Samples were sputter‐coated with gold using a Denton Desk V instrument for 30 s at 2 mA and imaged with 1–3 kV of accelerating voltage.

### 4.12 Micro‐ and Nano‐Pore Size Analysis

Fully formed micropores (50 count) were selected across 4–5 in‐plane SEM images (∼400–500x magnification) and measured with ImageJ for each of the various hydrogels. The scale was set using the “Set Scale” function and the rectangle tool to match that of the SEM image, and the pores were measured using the straight‐line tool to find their X and Y lengths. The X measurement spanned the shorter distance and bisected the pore into equal halves, while the Y measurement was perpendicular to this initial measurement and spanned the longer distance. The pore size measurements were collated into Excel for analysis by hydrogel design, including average cell size and standard deviation. Similarly, the fully formed nanopores (a minimum of 10 counts for the 3WJ‐Lo and 3WJ‐Lm and 7 counts for the 3WJ‐Sh) were selected in the AFM images and their dimensions measured with ImageJ [77] as for the micro‐pores (*n* = 1 sample for each type of hydrogel).

### 4.13 Rheology Measurements

Nanostructures were folded at the indicated molar concentrations in 1x folding buffer. Identical complementary structures were mixed on the Anton Paar MCR302 rheometer plate via pipette prior to rheology measurements. Rheology was conducted with a parallel plate setup (non‐sandblasted Peltier plate) using the PP08/S measuring system (97676; Anton Paar), and measurements were taken at a strain of 1%. Measurements were taken after mixing and then again after a heating and cooling cycle. The heating cycle increased linearly from 25°C to 55°C over 5 min with a 2‐min hold at 55°C followed by a cooling ramp to 25°C over the course of 5 min. The hydrogel was allowed to set for 5 min at 25°C before the second set of rheology measurements were taken.

### 4.14 Printing Tests

Prints were conducted at an ambient temperature ranging from 20°C–23°C using an Allevi 3 extrusion 3D printer (Allevi, Inc.). Initial line tests were performed to identify ideal printing parameters (Figure S33); the test indicated ideal parameters of 68 pounds PSI pressure with a 1‐inch, 30‐gauge nozzle. Subsequent tests were performed with rectilinear infill, printing a cube of 2 cm × 2 cm × 1 cm, as well as “GMU” block letters with one infill per layer. Rectilinear cube prints were inspected for cohesion and integrity to print vertically without deformation. Subsequently, the material was recollected, heated, recentrifuged, and reprinted (Figure 5e). “GMU” logo block letters were printed to identify the material's ability to support its own weight with only a single layer infill per Z stack, as well as the ability to manipulate in 90° corners and sweeping curves (Figure 5f).

### 4.15 Implementation of a DNA Strand Release System in the Hydrogel

The functionalization of the DNA hydrogels was assessed by quantifying the release of a quencher from the HEX‐modified DNA motifs (1 µm) and measuring the increase in fluorescence intensity for each fluorophore after the addition of a trigger strand (2:1 trigger:motif) at 40°C using the ‘Q’ quantitative polymerase chain reaction (qPCR) machine (Quantabio). The protocol was set to run for 60 cycles with 120 s of cycling time after a 5‐min hold period, wherein the cycling time increased 20 s per cycle (e.g., 140 s of cycling time for cycle two and 160 s for cycle three) for a total run time of ∼12 h. The excitation for HEX was 540 nm, and the emission was recorded at 570 nm. The release was normalized against the positive control, which was the DNA motif without the quencher, and all the values are reported as the mean ± standard deviation.

### 4.16 Hydrogel Mechanical Stability Assay

The mechanical stability of the 3WJ‐Sh hydrogels (60 µm) was determined with frequency sweeps at the indicated post‐assembly timepoints (0, 4, and 5 days) as previously described in the Rheology Measurements section. Between each measurement, the hydrogels were stored at 4°C.

### 4.17 Statistical Analysis and Reproducibility

All tests were performed in triplicates and all values were reported as the mean ± standard deviation, unless otherwise indicated. For the DLS, the pore size measurements and the rheology analyses, two‐tailed Student *t*‐tests were conducted assuming two samples of equal variance. *p*‐values were reported with specified thresholds for significance or non‐significance. All statistical analyses were conducted with Excel and reported in supplementary tables.

## Author Contributions

R.V.: conceptualization; D.V.S., S.J., R.V.: methodology; D.V.S., A.B.C., P.M.T., E.O.T., K.A.H., J.B., R.C.S., C.R.F., H.G.M., C.H., R.V.: investigation; D.V.S., R.V.original draft preparation; all authors: writing: review and editing; X.Y., S.J., R.V.: supervision; D.V.S., X.Y., S.J., R.V.: funding acquisition. All authors have read and agreed to the published version of the manuscript.

## Conflicts of Interest

The authors declare no conflicts of interest.

## Supporting information




**Supporting file**: advs73987‐sup‐0001‐SuppMat.docx

## Data Availability

Data are available upon reasonable request to the corresponding author.
