# Supplementary material for: Design and Characterization of DX‐Tile DNA Nanostar‐Based Hydrogels
Source: Adv Sci (Weinh). 2026 Feb 10;13(18):e14506. doi: 10.1002/advs.202514506 (PMC13042906; doi:10.1002/advs.202514506)
Supplement: Supplementary file 1 — Supporting file: advs73987‐sup‐0001‐SuppMat.docx [file ADVS-13-e14506-s001.docx]

Supporting Information

***Design and Characterization of DX-Tile DNA Nanostar-Based Hydrogels***

Dylan V. Scarton, Alessandra B. Coogan, Peter M. Touma, Eray O. Tulun, Katie A. Harrison, Jack Buchen, Richard C. Steiner, Christopher R. Fellin, Hunter G. Mason, Chih-Hsiang Hu, Sally Farag, Xiaoning Yuan, Shailly Jariwala, Remi Veneziano*

D.V. Scarton

Interdisciplinary Program in Neuroscience, College of Science, George Mason University, Fairfax, VA, USA

A.B. Coogan, P.M. Touma, E.O. Tulun, K.A. Harrison, CH. Hu, Sally Farag, R. Veneziano

Department of Bioengineering, College of Engineering and Computing, George Mason University, Fairfax, VA, USA

E-mail: [rvenezia@gmu.edu](mailto:rvenezia@gmu.edu)

D.V. Scarton, A.B. Coogan, P.M. Touma, E.O. Tulun, K.A. Harrison, CH. Hu, Sally Farag, R. Veneziano

Institute for Advanced Biomedical Research, George Mason University, Manassas, VA, USA

J. Buchen, R. C. Steiner, C. R. Fellin, S. Jariwala

Henry Jackson Foundation for the Advancement of Military Medicine, Inc., Bethesda, MD, USA

J. Buchen, R. C. Steiner, C. R. Fellin, X. Yuan, S. Jariwala

Department of Physical Medicine and Rehabilitation, Center for Rehabilitation Sciences Research, Uniformed Services University of the Health Sciences, Bethesda, MD, USA

H.G. Mason

School of System Biology, College of Science, George Mason University, Manassas, VA, USA

H.G. Mason

Center for Infectious Diseases, George Mason University, Manassas, VA, USA

**Table S1. DNA sequences for double-crossover (DX)-tile three-way junction (3WJ) motif with regular arm and blunt (Bl), short (Sh), long (Lo), or long mismatch (Lm) linkers, including variants with one (X) and two (XX) additional crossovers.** Regions highlighted in orange represents hybridizing sticky ends and **'** indicate strands used for the complementary motifs.

| **Component**  **(xMolar Ratio)** | **Strand** | **Sequence** |
| --- | --- | --- |
| **Core (x1)** | 1 | GGAAGTCTAGACAGTAGCGGCTTCCGCAAGATTTCACCAGGG |
| **Core (x1)** | 2 | GGAAGTCTAGCGATATAAATTTGTCGGGACTTTTCACCAGGG |
| **Core (x1)** | 3 | GGAAGTCTAGCGTAAGTCTCGGGGGTAGGAATTTCACCAGGG |
| **Core (x1)** | 4 | CGGAATTTGCCGCTACTGTAGTCCCGACATTTAATTTATATCGTTCCTACCCCTTTCGAGACTTACGTCTTG |
| **Arm (x3)** | 5(X) | AGGCACGGACCCCTGGTGAAACTAGACTTCCCCCTACATTTT |
| **Arm (x3)** | 5XX | ACCCCTGGTGAAACTAGACTTCCCCCTACATTTTAGGCACGG |
| **Arm (x3)** | 6 | AATCCCAGCCCGCTATGGACGTTGGTCTACGCGACCCCTCCG |
| **Arm (x3)** | 6X | GACCCCTCCGAATCCCAGCCCGCTATGGACGTTGGTCTACGC |
| **Arm (x3)** | 6XX | TATGGACGTTGGTCTACGCGACCCCTCCGAATCCCAGCCCGC |
| **Bl Linker (x3)** | 7 | CGTCCATAGCGGGCTGGGATTAAAATGTAGGG |
| **Bl Linker (x3)** | 8 | GTCCGTGCCTCGGAGGGGTCGCGTAGACCAA |
| **Sh Linker (x3)** | 7 | AGACATCCGTCCATAGCGGGCTGGGATTAAAATGTAGGG |
| **Sh Linker (x3)** | 8 | GTCCGTGCCTCGGAGGGGTCGCGTAGACCAACCTGCAC |
| **Sh Linker (x3)** | 7' | GATGTCTCGTCCATAGCGGGCTGGGATTAAAATGTAGGG |
| **Sh Linker (x3)** | 8' | GTCCGTGCCTCGGAGGGGTCGCGTAGACCAAGTGCAGG |
| **Lo Linker (x3)** | 7 | GAATTAATATATATAGACATCCGTCCATAGCGGGCTGGGATTAAAATGTAGGG |
| **Lo Linker (x3)** | 8 | GTCCGTGCCTCGGAGGGGTCGCGTAGACCAACCTGCACTTTGTTTGGCTAGG |
| **Lo Linker (x3)** | 7' | GATGTCTATATATATTAATTCCGTCCATAGCGGGCTGGGATTAAAATGTAGGG |
| **Lo Linker (x3)** | 8' | GTCCGTGCCTCGGAGGGGTCGCGTAGACCAACCTAGCCAAACAAAGTGCAGG |
| **Lm Linker (x3)** | 7 | GAATTAATTTTTTTAGACATCCGTCCATAGCGGGCTGGGATTAAAATGTAGGG |
| **Lm Linker (x3)** | 8 | GTCCGTGCCTCGGAGGGGTCGCGTAGACCAACCTGCACTTTTTTTGGCTAGG |
| **Lm Linker (x3)** | 7' | GATGTCTTTTTTTTTTAATTCCGTCCATAGCGGGCTGGGATTAAAATGTAGGG |
| **Lm Linker (x3)** | 8' | GTCCGTGCCTCGGAGGGGTCGCGTAGACCAACCTAGCCTTTTTTTGTGCAGG |

**Table S2.** **P-values for the DLS experiments in Figure 1b**.

| **Row** | **3WJ-Sh** | **3WJ-Lo** | **3WJ-Lm** |
| --- | --- | --- | --- |
| **3WJ-Sh** | N/A | 0.0694 | 0.0315 |
| **3WJ-Lo** | 0.0694 | N/A | 0.0854 |
| **3WJ-Lm** | 0.0315 | 0.0854 | N/A |

**Table S3.** **SEM micropore size measurements**.

| **Row** | **3WJ-Sh** | **3WJ-Lo** | **3WJ-Lm** | **3WJ-Bl** |
| --- | --- | --- | --- | --- |
| **60 µm** | 4.4 ± 0.2 µm | 5.0 ± 0.3 µm | 5.5 ± 0.2 µm | 5.8 ± 0.3 µm |
| **90 µm** | 4.8 ± 0.2 µm | 5.4 ± 0.3 µm | 5.7 ± 0.2 µm | 5.3 ± 0.3 µm |

**Table S4. P-values for the micropores size measurements in Figure 2b.**

| **Row** | **3WJ-Sh**  **(60 µm)** | **3WJ-Lm**  **(60 µm)** | **3WJ-Lo**  **(60 µm)** | **3WJ-Sh**  **(90 µm)** | **3WJ-Lm**  **(90 µm)** | **3WJ-Lo**  **(90 µm)** |
| --- | --- | --- | --- | --- | --- | --- |
| **3WJ-Sh**  **(60 µm)** | N/A | 0.0007 | 0.0861 | 0.1794 | N/A | N/A |
| **3WJ-Lm**  **(60 µm)** | 0.00073 | N/A | 0.1460 | N/A | 0.6013 | N/A |
| **3WJ-Lo**  **(60 µm)** | 0.0861 | 0.1460 | N/A | N/A | N/A | 0.3874 |
| **3WJ-Sh**  **(90 µm)** | 0.1794 | N/A | N/A | N/A | 0.0014 | 0.0966 |
| **3WJ-Lm**  **(90 µm)** | N/A | 0.6014 | N/A | 0.0014 | N/A | 0.3799 |
| **3WJ-Lo**  **(90 µm)** | N/A | N/A | 0.3874 | 0.0966 | 0.3799 | N/A |

**Table S5.** **Y motif sequences.** Regions highlighted in orange represents hybridizing sticky ends.

| **Component**  **(xMolar Ratio)** | **Strand** | **Sequence** |
| --- | --- | --- |
| **Y7** | | |
| **A Motif (x1)** | A1 | CAGAGCTGTCCGTGAGTGAGGCAGGATGGACGCCCGCGCTGATCCGTGA |
| **A Motif (x1)** | A2 | CAGAGCTTCACGGATCAGCGCGGGCGTCTAACTTCCTGGCAGGCCGACT |
| **A Motif (x1)** | A3 | CAGAGCTAGTCGGCCTGCCAGGAAGTTACATCCTGCCTCACTCACGGAC |
| **B Motif (x1)** | B1 | AGCTCTGGTCCGTGAGTGAGGCAGGATGGACGCCCGCGCTGATCCGTGA |
| **B Motif (x1)** | B2 | AGCTCTGTCACGGATCAGCGCGGGCGTCTAACTTCCTGGCAGGCCGACT |
| **B Motif (x1)** | B3 | AGCTCTGAGTCGGCCTGCCAGGAAGTTACATCCTGCCTCACTCACGGAC |
| **Y21** | | |
| **A Motif (x1)** | A1 | GAATTAATATATATAGACATCGTCCGTGAGTGAGGCAGGATGGACGCCCGCGCTGATCCGTGA |
| **A Motif (x1)** | A2 | GAATTAATATATATAGACATCTCACGGATCAGCGCGGGCGTCTAACTTCCTGGCAGGCCGACT |
| **A Motif (x1)** | A3 | GAATTAATATATATAGACATCAGTCGGCCTGCCAGGAAGTTACATCCTGCCTCACTCACGGAC |
| **B Motif (x1)** | B1 | GATGTCTATATATATTAATTCGTCCGTGAGTGAGGCAGGATGGACGCCCGCGCTGATCCGTGA |
| **B Motif (x1)** | B2 | GATGTCTATATATATTAATTCTCACGGATCAGCGCGGGCGTCTAACTTCCTGGCAGGCCGACT |
| **B Motif (x1)** | B3 | GATGTCTATATATATTAATTCAGTCGGCCTGCCAGGAAGTTACATCCTGCCTCACTCACGGAC |

**Table S6. Comparison of the motif concentration ([Motif]) and total DNA concentration ([DNA]) used for each tested 3WJ and Y motifs.** Total DNA concentrations were calculated using the total number of bases per motif. Among these tested motif concentrations, cells shaded in orange represent motif concentrations that did not form gels, while yellow shading represents partial gel formation and green represents definitive gel formation.

| **Y7 (147 bases)** | | **Y21 (189 bases)** | | **3WJ-Sh (681 bases)** | | | **3WJ-Lo/Lm (765 bases)** | |
| --- | --- | --- | --- | --- | --- | --- | --- | --- |
| **Motif**  **[µm]** | **DNA**  **[mm]** | **Motif**  **[µm]** | **DNA**  **[mm]** | **Motif**  **[µm]** | | **DNA**  **[mm]** | **Motif**  **[µm]** | **DNA**  **[mm]** |
| 37.5 | 5.51 | 30 | 5.67 | 15 | 10.22 | | 15 | 11.48 |
| 75 | 11.02 | 60 | 11.34 | 30 | 20.43 | | 30 | 22.95 |
| 150 | 22.05 | 120 | 22.68 | 50 | 34.05 | | 50 | 38.25 |
| 225 | 33.08 | 180 | 34.02 | 60 | 40.86 | | 60 | 45.90 |
|  | | | | 90 | 61.29 | | 90 | 68.85 |

**Table S7. P-values for the Table 1.**

| **Rows** | **3WJ-Sh**  **(30 µm)** | **3WJ-Sh**  **(60 µm)** | **3WJ-Sh**  **(90 µm)** | **3WJ-Lm**  **(30 µm)** | **3WJ-Lm**  **(60 µm)** | **3WJ-Lm**  **(90 µm)** | **3WJ-Lo**  **(30 µm)** | **3WJ-Lo**  **(60 µm)** | **3WJ-Lo**  **(90 µm)** |
| --- | --- | --- | --- | --- | --- | --- | --- | --- | --- |
| **3WJ-Sh**  **(30 µm)** | N/A | 0.0000 | 0.0000 | 0.0022 | N/A | N/A | 0.3902 | N/A | N/A |
| **3WJ-Sh**  **(60 µm)** | 0.0000 | N/A | 0.0000 | N/A | 0.0725 | N/A | N/A | 0.1420 | N/A |
| **3WJ-Sh**  **(90 µm)** | 0.0000 | 0.0000 | N/A | N/A | N/A | 0.1772 | N/A | N/A | 0.5402 |
| **3WJ-Lm**  **(30 µm)** | 0.0022 | N/A | N/A | N/A | 0.0014 | 0.0002 | 0.2480 | N/A | N/A |
| **3WJ-Lm**  **(60 µm)** | N/A | 0.0725 | N/A | 0.0014 | N/A | 0.0009 | N/A | 0.0431 | N/A |
| **3WJ-Lm**  **(90 µm)** | N/A | N/A | 0.1772 | 0.0002 | 0.0009 | N/A | N/A | N/A | 0.1967 |
| **3WJ-Lo**  **(30 µm)** | 0.3902 | N/A | N/A | 0.2480 | N/A | N/A | N/A | 0.0011 | 0.0003 |
| **3WJ-Lo**  **(60 µm)** | N/A | 0.1420 | N/A | N/A | 0.0431 | N/A | 0.0011 | N/A | 0.0021 |
| **3WJ-Lo**  **(90 µm)** | N/A | N/A | 0.5402 | N/A | N/A | 0.1967 | 0.0003 | 0.0021 | N/A |

**Table S8. Comparison of storage moduli at one hertz after heat cycling for 3WJ-Sh and 6WJ-Sh hydrogels.** For 3WJ (60 µm, 90 µm) and 6WJ (25 µm, 50 µm) hydrogels, the baseline storage modulus is compared with the storage modulus following the heat cycle protocol. Statistical analyses are available in Table S9.

| **motifs** | **3WJ-Sh (60 µm**) | | **3WJ-Sh (90 µm**) | | **6WJ-Sh (25 µm**) | | **6WJ-Sh (50 µm**) | |
| --- | --- | --- | --- | --- | --- | --- | --- | --- |
| **Conditions** | **Base** | **Heat** | **Base** | **Heat** | **Base** | **Heat** | **Base** | **Heat** |
| **G’ [Pa]** | 296 ± 6 | 224 ± 48 | 1010 ± 17 | 793 ± 87 | 118 ± 12 Pa | 29 ± 17 | 1266 ± 230 | 347 ± 29 |

**Table S9. P-values for the Table S8.**

| **Name** | **3WJ** | | | | **6WJ** | | | |
| --- | --- | --- | --- | --- | --- | --- | --- | --- |
| **Conditions** | **Base** | **Heat** | **Base** | **Heat** | **Base** | **Heat** | **Base** | **Heat** |
| **[motifs]** | **60 µm** | **60 µm** | **90 µm** | **90 µm** | **25 µm** | **25 µm** | **50 µm** | **50 µm** |
| **Base** | N/A | 0.1032 | N/A | 0.0245 | N/A | 0.0036 | N/A | 0.0050 |
| **Heat** | 0.1032 | N/A | 0.0245 | N/A | 0.0036 | N/A | 0.0050 | N/A |

**Table S10. DNA sequences for DX-tile 3WJ motifs with short arm variants and 7-bp overhang.** Crossover (X) location indicated by name and represented in corresponding schematic: Repeating X-Out (***Figure S1bi***) or X-In (***Figure S1bii***) and Non-Repeating X-In (***Figure S1biii***). Regions highlighted in orange represents hybridizing sticky ends.

| **Component (xMolar Ratio)** | **Strand** | | **Sequence** | |
| --- | --- | --- | --- | --- |
|  | |  | | **Repeating Arms** |
| **Shared Strands (x1)** | | 1 | | AGACATCCGTCCATAGCGGGCTGGGATTCGTAAGTCTCGGGGGTAGGAACGGAGGGGTCGCGTAGACCAACCTGCAC |
| **Shared Strands (x1)** | | 1’ | | GATGTCTCGTCCATAGCGGGCTGGGATTCGTAAGTCTCGGGGGTAGGAACGGAGGGGTCGCGTAGACCAAGTGCAGG |
| **Shared Strands (x1)** | | 2 | | AGACATCCGTCCATAGCGGGCTGGGATTCGATATAAATTTGTCGGGACTCGGAGGGGTCGCGTAGACCAACCTGCAC |
| **Shared Strands (x1)** | | 2’ | | GATGTCTCGTCCATAGCGGGCTGGGATTCGATATAAATTTGTCGGGACTCGGAGGGGTCGCGTAGACCAAGTGCAGG |
| **Shared Strands (x1)** | | 3 | | AGACATCCGTCCATAGCGGGCTGGGATTACAGTAGCGGCTTCCGCAAGACGGAGGGGTCGCGTAGACCAACCTGCAC |
| **Shared Strands (x1)** | | 3’ | | GATGTCTCGTCCATAGCGGGCTGGGATTACAGTAGCGGCTTCCGCAAGACGGAGGGGTCGCGTAGACCAAGTGCAGG |
| **Shared Strands (x1)** | | 4 | | CGGAATTTGCCGCTACTGTAGTCCCGACATTTAATTTATATCGTTCCTACCCCTTTCGAGACTTACGTCTTG |
| **X-Out Strand (x3)** | | 5 | | AATCCCAGCCCGCTATGGACGTTGGTCTACGCGACCCCTCCG |
| **X-In Strand (x3)** | | 5 | | TTGGTCTACGCGACCCCTCCGAATCCCAGCCCGCTATGGACG |
|  | |  | | **Non-Repeating Arms** |
| **X-In Strands (x1)** | | 1 | | AGACATCCGCACATTGTTGGAATCAGGCCGTAAGTCTCGGGGGTAGGAATTTCACCAGGGGTCCGTGCCTCCTGCAC |
| **X-In Strands (x1)** | | 1’ | | GATGTCTCGCACATTGTTGGAATCAGGCCGTAAGTCTCGGGGGTAGGAATTTCACCAGGGGTCCGTGCCTGTGCAGG |
| **X-In Strands (x1)** | | 2 | | AGACATCTTAGAAGCGGTGACTTGACCTACAGTAGCGGCTTCCGCAAGACCAGTAAGTCGTGGCGCCATCCCTGCAC |
| **X-In Strands (x1)** | | 2’ | | GATGTCTTTAGAAGCGGTGACTTGACCTACAGTAGCGGCTTCCGCAAGACCAGTAAGTCGTGGCGCCATCGTGCAGG |
| **X-In Strands (x1)** | | 3 | | AGACATCAAAATGTAGGGGGAAGTCTAGCGATATAAATTTGTCGGGACTCGTATTGCCCAGTGTGCGGTACCTGCAC |
| **X-In Strands (x1)** | | 3’ | | GATGTCTAAAATGTAGGGGGAAGTCTAGCGATATAAATTTGTCGGGACTCGTATTGCCCAGTGTGCGGTAGTGCAGG |
| **X-In Strands (x1)** | | 4 | | TCTTGCGGAATTTGCCGCTACTGTAGTCCCGACATTTAATTTATATCGTTCCTACCCCTTTCGAGACTTACG |
| **X-In Strands (x1)** | | 5 | | GATGGCGCCACGACTTACTGGGCCTGATTCCAACAATGTGCG |
| **X-In Strands (x1)** | | 6 | | TACCGCACACTGGGCAATACGAGGTCAAGTCACCGCTTCTAA |
| **X-In Strands (x1)** | | 7 | | AGGCACGGACCCCTGGTGAAACTAGACTTCCCCCTACATTTT |

**Table S11. DNA sequences for DX-tile 3WJ motifs with long arm and Sh linker.** Regions highlighted in orange represents hybridizing sticky ends.

| **Component**  **(xMolar Ratio)** | **Strand** | **Sequence** |
| --- | --- | --- |
| **Core (x1)** | 1 | GGAAGTCTAGACAGTAGCGGCTTCCGCAAGATTTCACCAGGG |
| **Core (x1)** | 2 | GGAAGTCTAGCGATATAAATTTGTCGGGACTTTTCACCAGGG |
| **Core (x1)** | 3 | GGAAGTCTAGCGTAAGTCTCGGGGGTAGGAATTTCACCAGGG |
| **Core (x1)** | 4 | CGGAATTTGCCGCTACTGTAGTCCCGACATTTAATTTATATCGTTCCTACCCCTTTCGAGACTTACGTCTTG |
| **Long Arm (x3)** | 5 | AGTTGGCACACCCTGGTGAAACTAGACTTCCAGACGGTGCGA |
| **Long Arm (x3)** | 6 | GCCGATGTCCGCGCCAGCTGGGAAGCAAACTGGGGAACGGAT |
| **Long Arm (x3)** | 7 | TCACTACGTCGACCCCGCCTCTGAGGTGCCCCGCGGCCACAC |
| **Sh Linker (x3)** | 8 | TGTGCCAACTCCAGCTGGCGCGGACATCGGCGTGTGGCCGCGGGGCACCTCACCTGCAC |
| **Sh Linker (x3)** | 9 | AGACATCGAGGCGGGGTCGACGTAGTGAATCCGTTCCCCAGTTTGCTTCTCGCACCGTCT |
| **Sh Linker (x3)** | 8’ | TGTGCCAACTCCAGCTGGCGCGGACATCGGCGTGTGGCCGCGGGGCACCTCAGT GCAGG |
| **Sh Linker (x3)** | 9’ | GATGTCTGAGGCGGGGTCGACGTAGTGAATCCGTTCCCCAGTTTGCTTCTCGCACCGTCT |

**Table S12. DX-tile six-way junction (6WJ) sequences.** Linker sequences are the same as for the 3WJ. Regions highlighted in orange represents hybridizing sticky ends.

| **Component**  **(xMolar Ratio)** | **Strand** | **Sequence** |
| --- | --- | --- |
| **Core (x1)** | 1 | AGCTATCCCCTTTTTCGAGACTTACGCCTCTGGAGTTTTTTCTTGCCAGGCA |
| **Core (x1)** | 2 | GGAAGTCTAGCGTAAGTCTCGGGGGATAGCTTTTCACCAGGG |
| **Core (x1)** | 3 | GGAAGTCTAGGACGCCAGGAGTATATATACGTTTCACCAGGG |
| **Core (x1)** | 4 | TTCCTACCCCTTTTTAAACTACTGTACGTATATATATTTTTCTCCTGGCGTC |
| **Core (x1)** | 5 | GGAAGTCTAGTGCCTGGCAAGACTCCAGAGGTTTCACCAGGG |
| **Core (x1)** | 6 | TCTTGCGGAATTTTTGCCGCTACTGTAGTCACGACATTTTTAATTTATATCG |
| **Core (x1)** | 7 | GGAAGTCTAGACAGTAGCGGCTTCCGCAAGATTTCACCAGGG |
| **Core (x1)** | 8 | GGAAGTCTAGCGATATAAATTTGTCGTGACTTTTCACCAGGG |
| **Core (x1)** | 9 | GGAAGTCTAGTACAGTAGTTTGGGGTAGGAATTTCACCAGGG |
| **Arm (x6)** | 10 | AGGCACGGACCCCTGGTGAAACTAGACTTCCCCCTACATTTT |
| **Arm (x6)** | 11X | GACCCCTCCGAATCCCAGCCCGCTATGGACGTTGGTCTACGC |
| **Arm (x6)** | 11ON | AATCCCAGCCCGCTATGGACGTTGGTCTACGCGACCCCTCCG |
| **Bl Linker (x6)** | 12 | CGTCCATAGCGGGCTGGGATTAAAATGTAGGG |
| **Bl Linker (x6)** | 13 | GTCCGTGCCTCGGAGGGGTCGCGTAGACCAA |
| **Sh Linker (x6)** | 12 | AGACATCCGTCCATAGCGGGCTGGGATTAAAATGTAGGG |
| **Sh Linker (x6)**  **Sh Linker (x6)** | 13 | GTCCGTGCCTCGGAGGGGTCGCGTAGACCAACCTGCAC |
| **Sh Linker (x6)** | 12' | GATGTCTCGTCCATAGCGGGCTGGGATTAAAATGTAGGG |
| **Lo Linker (x6)** | 13' | GTCCGTGCCTCGGAGGGGTCGCGTAGACCAAGTGCAGG |
| **Lo Linker (x6)** | 12 | GAATTAATATATATAGACATCCGTCCATAGCGGGCTGGGATTAAAATGTAGGG |
| **Lo Linker (x6)** | 13 | GTCCGTGCCTCGGAGGGGTCGCGTAGACCAACCTGCACTTTGTTTGGCTAGG |
| **Lo Linker (x6)** | 12' | GATGTCTATATATATTAATTCCGTCCATAGCGGGCTGGGATTAAAATGTAGGG |
| **Lo Linker (x6)** | 13' | GTCCGTGCCTCGGAGGGGTCGCGTAGACCAACCTAGCCAAACAAAGTGCAGG |
| **Lm Linker (x6)** | 12 | GAATTAATTTTTTTAGACATCCGTCCATAGCGGGCTGGGATTAAAATGTAGGG |
| **Lm Linker (x6)** | 13 | GTCCGTGCCTCGGAGGGGTCGCGTAGACCAACCTGCACTTTTTTTGGCTAGG |
| **Lm Linker (x6)** | 12' | GATGTCTTTTTTTTTTAATTCCGTCCATAGCGGGCTGGGATTAAAATGTAGGG |
| **Lm Linker (x6)** | 13' | GTCCGTGCCTCGGAGGGGTCGCGTAGACCAACCTAGCCTTTTTTTGTGCAGG |

**Table S13. P-values for the Table 2**

| **Rows** | **3WJ-**  **Sh**  **(50 µm)** | **3WJ-**  **Lm**  **(50 µm)** | **3WJ-**  **Lo**  **(50 µm)** | **4WJ-**  **Sh**  **(50 µm)** | **4WJ-**  **Lm**  **(50 µm)** | **4WJ-**  **Lo**  **(50 µm)** | **5WJ-**  **Sh**  **(50 µm)** | **5WJ-**  **Lm**  **(50 µm)** | **5WJ-**  **Lo**  **(50 µm)** | **6WJ-**  **Sh**  **(50 µm)** | **6WJ-**  **Lm**  **(50 µm)** | **6WJ-**  **Lo**  **(50 µm)** |
| --- | --- | --- | --- | --- | --- | --- | --- | --- | --- | --- | --- | --- |
| **3WJ-**  **Sh**  **(50 µm)** | N/A | 0.2227 | 0.5974 | 0.0030 | 0.0012 | 0.0001 | 0.0001 | 0.0000 | 0.0013 | 0.0024 | 0.0003 | 0.0000 |
| **3WJ-**  **Lm**  **(50 µm)** | 0.2227 | N/A | 0.2618 | 0.0027 | 0.0013 | 0.0001 | 0.0001 | 0.0000 | 0.0012 | 0.0022 | 0.0003 | 0.0000 |
| **3WJ-**  **Lo**  **(50 µm)** | 0.5974 | 0.2618 | N/A | 0.0162 | 0.0084 | 0.0008 | 0.0003 | 0.0001 | 0.0019 | 0.0027 | 0.0004 | 0.0000 |
| **4WJ-**  **Sh**  **(50 µm)** | 0.0030 | 0.0027 | 0.0162 | N/A | 0.5079 | 0.0061 | 0.0010 | 0.0002 | 0.0060 | 0.0049 | 0.0008 | 0.0000 |
| **4WJ-**  **Lm**  **(50 µm)** | 0.0012 | 0.0013 | 0.0084 | 0.5079 | N/A | 0.0084 | 0.0011 | 0.0002 | 0.0072 | 0.0054 | 0.0009 | 0.0000 |
| **4WJ-**  **Lo**  **(50 µm)** | 0.0001 | 0.0001 | 0.0008 | 0.0061 | 0.0084 | N/A | 0.0071 | 0.0006 | 0.0349 | 0.0103 | 0.0018 | 0.0000 |
| **5WJ-**  **Sh**  **(50 µm)** | 0.0001 | 0.0001 | 0.0003 | 0.0010 | 0.0011 | 0.0071 | N/A | 0.2658 | 0.7614 | 0.0338 | 0.0094 | 0.0007 |
| **5WJ-**  **Lm**  **(50 µm)** | 0.0000 | 0.0000 | 0.0001 | 0.0002 | 0.0002 | 0.0006 | 0.2658 | N/A | 0.7471 | 0.0440 | 0.0114 | 0.0001 |
| **5WJ-**  **Lo**  **(50 µm)** | 0.0013 | 0.0012 | 0.0019 | 0.0060 | 0.0072 | 0.0349 | 0.7614 | 0.7471 | N/A | 0.0488 | 0.0208 | 0.0120 |
| **6WJ-**  **Sh**  **(50 µm)** | 0.0024 | 0.0022 | 0.0027 | 0.0049 | 0.0054 | 0.0103 | 0.0338 | 0.0440 | 0.0488 | N/A | 0.7426 | 0.3524 |
| **6WJ-**  **Lm**  **(50 µm)** | 0.0003 | 0.0003 | 0.0004 | 0.0008 | 0.0009 | 0.0018 | 0.0094 | 0.0114 | 0.0208 | 0.7426 | N/A | 0.3094 |
| **6WJ-**  **Lo**  **(50 µm)** | 0.0000 | 0.0000 | 0.0000 | 0.0000 | 0.0000 | 0.0000 | 0.0007 | 0.0001 | 0.0120 | 0.3524 | 0.3094 | N/A |

**Table S14. DX-tile four-way junction (4WJ) sequences.** Linker sequences are the same as for the 3WJ. Regions highlighted in orange represents hybridizing sticky ends.

| **Component**  **(xMolar Ratio)** | **Strand** | **Sequence** |
| --- | --- | --- |
| **Core (x1)** | 1 | GGAAGTCTAGCGATATAAATTTGTCGTGACTTTTCACCAGGG |
| **Core (x1)** | 2 | GGAAGTCTAGTACAGTAGTTTGGGGTAGGAATTTCACCAGGG |
| **Core (x1)** | 3 | GGAAGTCTAGTGCCTGGCAAGACTCCAGAGGTTTCACCAGGG |
| **Core (x1)** | 4 | GGAAGTCTAGACAGTAGCGGCTTCCGCAAGATTTCACCAGGG |
| **Core (x1)** | 5 | TCTTGCGGAATTTTGCCGCTACTGTAGTCACGACATTTTGAGCCCTGTCC |
| **Core (x1)** | 6 | GCTCCCACGCTTTTCGAGACTTACGCCTCTGGAGTTTTTCTTGCCAGGCA |
| **Arm (x4)** | 7 | AGGCACGGACCCCTGGTGAAACTAGACTTCCCCCTACATTTT |
| **Arm (x4)** | 8X | GACCCCTCCGAATCCCAGCCCGCTATGGACGTTGGTCTACGC |
| **Arm (x4)** | 8ON | AATCCCAGCCCGCTATGGACGTTGGTCTACGCGACCCCTCCG |
| **Sh Linker (x4)** | 9 | AGACATCCGTCCATAGCGGGCTGGGATTAAAATGTAGGG |
| **Sh Linker (x4)** | 10 | GTCCGTGCCTCGGAGGGGTCGCGTAGACCAACCTGCAC |
| **Sh Linker (x4)** | 9' | GATGTCTCGTCCATAGCGGGCTGGGATTAAAATGTAGGG |
| **Sh Linker (x4)** | 10' | GTCCGTGCCTCGGAGGGGTCGCGTAGACCAAGTGCAGG |
| **Lo Linker (x4)** | 9 | GAATTAATATATATAGACATCCGTCCATAGCGGGCTGGGATTAAAATGTAGG |
| **Lo Linker (x4)** | 10 | GTCCGTGCCTCGGAGGGGTCGCGTAGACCAACCTGCACTTTGTTTGGCTAGG |
| **Lo Linker (x4)** | 9' | GATGTCTATATATATTAATTCCGTCCATAGCGGGCTGGGATTAAAATGTAGGG |
| **Lo Linker (x4)** | 10' | GTCCGTGCCTCGGAGGGGTCGCGTAGACCAACCTAGCCAAACAAAGTGCAGG |
| **Lm Linker (x4)** | 9 | GAATTAATTTTTTTAGACATCCGTCCATAGCGGGCTGGGATTAAAATGTAGGG |
| **Lm Linker (x4)** | 10 | GTCCGTGCCTCGGAGGGGTCGCGTAGACCAACCTGCACTTTTTTTGGCTAGG |
| **Lm Linker (x4)** | 9' | GATGTCTTTTTTTTTTAATTCCGTCCATAGCGGGCTGGGATTAAAATGTAGGG |
| **Lm Linker (x4)** | 10' | GTCCGTGCCTCGGAGGGGTCGCGTAGACCAACCTAGCCTTTTTTTGTGCAGG |

**Table S15. DX-tile five-way junction (5WJ) sequences.** Linker sequences are the same as for the 3WJ. Regions highlighted in orange represents hybridizing sticky ends.

| **Component**  **(xMolar Ratio)** | **Strand** | **Sequence** |
| --- | --- | --- |
| **Core (x1)** | 1 | GGAAGTCTAGCGATATAAATTTGTCGTGACTTTTCACCAGGG |
| **Core (x1)** | 2 | GGAAGTCTAGTACAGTAGTTTGGGGTAGGAATTTCACCAGGG |
| **Core (x1)** | 3 | GGAAGTCTAGGACGCCAGGAGTATATATACGTTTCACCAGGG |
| **Core (x1)** | 4 | GGAAGTCTAGCGTAAGTCTCGGGGGATAGCTTTTCACCAGGG |
| **Core (x1)** | 5 | GGAAGTCTAGTGCCTGGCAAGACTCCAGAGGTTTCACCAGGG |
| **Core (x1)** | 6 | CGTATATATATTTTCTCCTGGCGTCAGCTATCCCCTTTTCGAGACTTACG |
| **Core (x1)** | 7 | CCTCTGGAGTTTTTCTTGCCAGGCAAGTCACGACATTTTAATTTATATCGTTCCTACCCCTTTTAAACTACTGTA |
| **Arm (x5)** | 8 | AGGCACGGACCCCTGGTGAAACTAGACTTCCCCCTACATTTT |
| **Arm (x5)** | 9X | GACCCCTCCGAATCCCAGCCCGCTATGGACGTTGGTCTACGC |
| **Arm (x5)** | 9ON | AATCCCAGCCCGCTATGGACGTTGGTCTACGCGACCCCTCCG |
| **Sh Linker (x5)** | 10 | AGACATCCGTCCATAGCGGGCTGGGATTAAAATGTAGGG |
| **Sh Linker (x5)** | 11 | GTCCGTGCCTCGGAGGGGTCGCGTAGACCAACCTGCAC |
| **Sh Linker (x5)** | 10' | GATGTCTCGTCCATAGCGGGCTGGGATTAAAATGTAGGG |
| **Sh Linker (x5)** | 11' | GTCCGTGCCTCGGAGGGGTCGCGTAGACCAAGTGCAGG |
| **Lo Linker (x5)** | 10 | GAATTAATATATATAGACATCCGTCCATAGCGGGCTGGGATTAAAATGTAGG |
| **Lo Linker (x5)** | 11 | GTCCGTGCCTCGGAGGGGTCGCGTAGACCAACCTGCACTTTGTTTGGCTAGG |
| **Lo Linker (x5)** | 10' | GATGTCTATATATATTAATTCCGTCCATAGCGGGCTGGGATTAAAATGTAGGG |
| **Lo Linker (x5)** | 11' | GTCCGTGCCTCGGAGGGGTCGCGTAGACCAACCTAGCCAAACAAAGTGCAGG |
| **Lm Linker (x5)** | 10 | GAATTAATTTTTTTAGACATCCGTCCATAGCGGGCTGGGATTAAAATGTAGGG |
| **Lm Linker (x5)** | 11 | GTCCGTGCCTCGGAGGGGTCGCGTAGACCAACCTGCACTTTTTTTGGCTAGG |
| **Lm Linker (x5)** | 10' | GATGTCTTTTTTTTTTAATTCCGTCCATAGCGGGCTGGGATTAAAATGTAGGG |
| **Lm Linker (x5)** | 11' | GTCCGTGCCTCGGAGGGGTCGCGTAGACCAACCTAGCCTTTTTTTGTGCAGG |

**Table S16. Functional strand release profile in 3WJ-Lm and 6WJ-Lm hydrogels.** Summary results of the functional strand release system with hexachloro-fluorescein (HEX) fluorophore and IowaBlack quencher as cargo model, showing complete saturation (*Plateau*), release rate (*k, hr^-1^ and min^-1^*), r^2^ value of linear regression, and half-time course (*hr*) (3WJ *n* = 3 and 6WJ *n* = 7 independent samples/group). 3WJ and 6WJ hydrogels were made with the Sh linker at comparable concentrations of 60 µm and 30 µm, respectively.

| **3WJ-Lm at 60µm** | | | **6WJ-Lm at 30µm** | | |
| --- | --- | --- | --- | --- | --- |
| **Plateau** | 1022.5 ± 76.9 | nM | **Plateau** | 1021.9 ± 256.3 | nM |
| **k** | 0.54 ± 0.25 | hr^-1^ | **k** | 0.44 ± 0.26 | hr^-1^ |
|  | 32.15 | min^-1^ |  | 26.47 | min^-1^ |
| **r^2^** | 0.99 | --- | **r^2^** | 0.99 | --- |
| **Half-Time** | 1.29 ± 0.79 | hr | **Half-Time** | 1.57 ± 1.41 | hr |


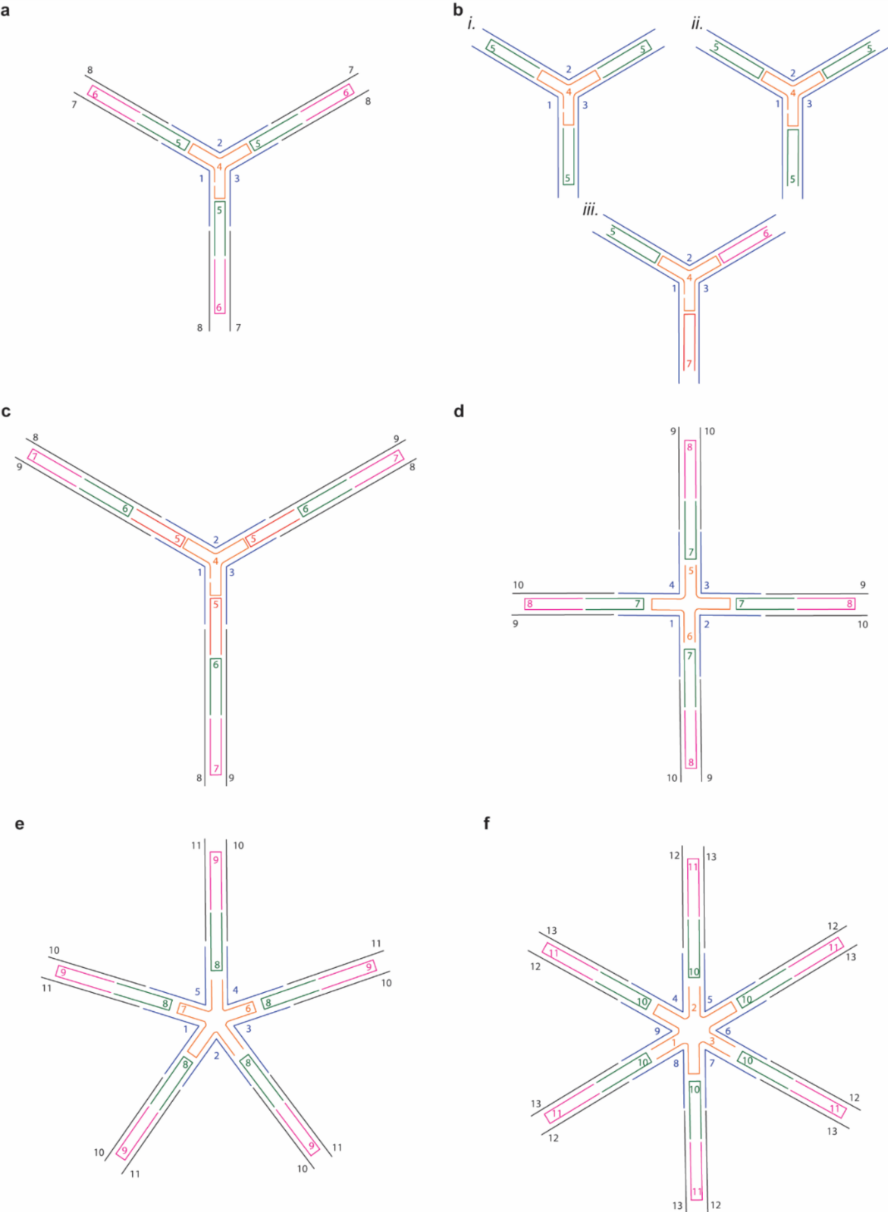


**Figure S1.** **Strand numbering schematic for the three DX-tile 3WJ motifs and the DX-tile 6WJ motif used in this study, all shown here with the Sh linker**. **a)** 3WJ with regular arms (53 bp). **b)** three variations of the 3WJ with short arms (32 bp) for crossover (X) locations on the repeating internal arm strand placed outside (*X-out, i*) or inside (*X-in, ii*) and the non-repeating internal arm strand placed inside (*X-in, iii*). **c)** 3WJ with long arms (74 bp). **d)** 4WJ with regular arms (53 bp). **e)** 5WJ with regular arms (53 bp). **f)** 6WJ with regular arms (53 bp).


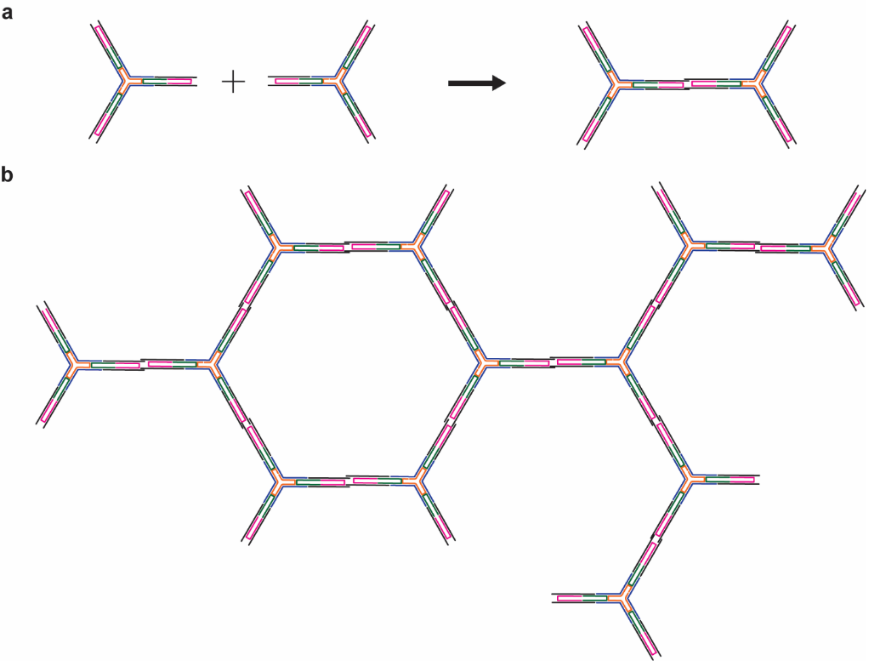


**Figure S2.** **Schematic of the putative DNA hydrogel mesh formation with 3WJ structural motifs.** **a)** 3WJ regular arms motifs with DNA linkers (linkers of A motif hybridize to linkers of B motif) to form a dimer. **b)** Potential graphic representation of a multi-motifs network.


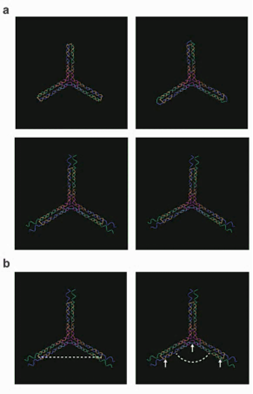
[Text Wrapping Break]

**Figure S3.** **Three dimensional representations and theoretical measurements of different 3WJ motif designs used in this study.** **a)** 3WJ motifs with Bl, Sh, Lo, and Lm linkers (*clockwise from top left*). **b)** Estimation of the distance (*left*) and angle (*right*) between each arm on the 3WJ motif, shown here with the Lo linker. White arrows denote specific atoms selected for measurements.


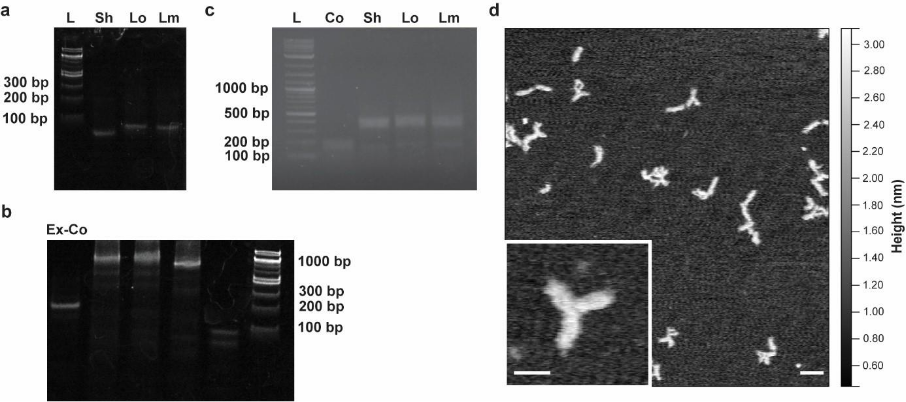


**Figure S4.** **Characterization of the 3WJ motifs with regular arms.** (**a**) PAGE characterization of the arms with linkers Short (Sh), Long (Lo), Long mismatch (Lm). (**b**) PAGE characterization of the 3WJ motifs from the core to the fully assembled structures with linkers. Core (Co) and extended Core (Ext-Co) components shown along fully folded motifs with Sh, Lo, and Lm linker types. (**c**) Agarose gel characterization of the 3WJ motifs from the core to the fully assembled structures with Sh, Lo, and Lm linker types. (**d**) Representative atomic force microscopy image showing assembly of 3WJ-Lo motifs (scale bar: 50 nm) and of a single 3WJ-Lo motif (inset, scale bar: 20 nm).


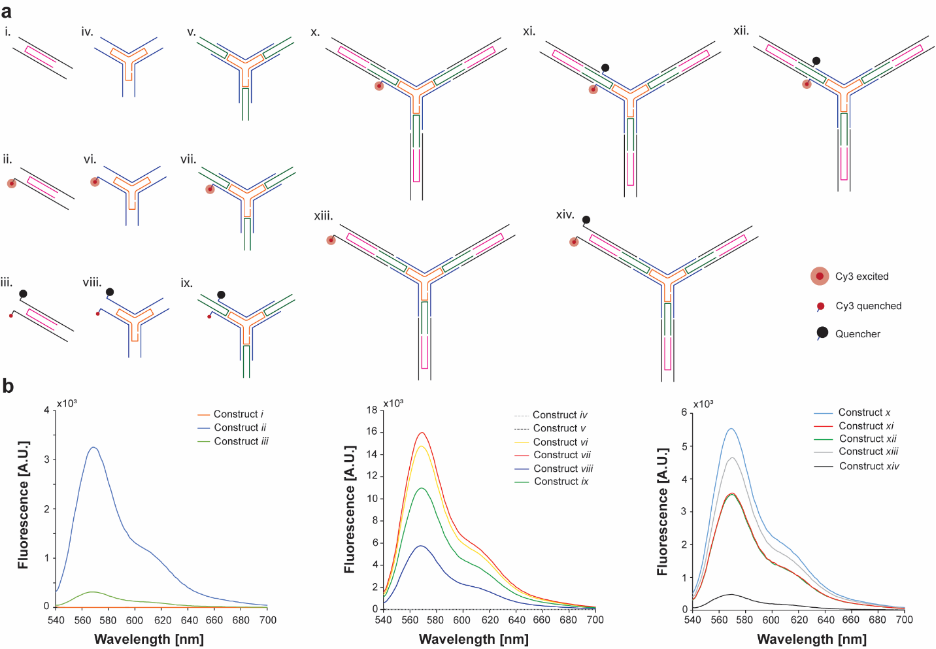


**Figure S5.** **FRET assay with Cy3/IowaBlack dye/quencher pair to monitor sequential formation of the DX-tile motifs.** **a)** schematic representation of the different constructs tested and the location of the FRET pairs. **b)** Fluorescence measurements of the different constructs after annealing. The constructs were excited at 520 nm wavelength and the emission was recorded from 540 nm to 700 nm.


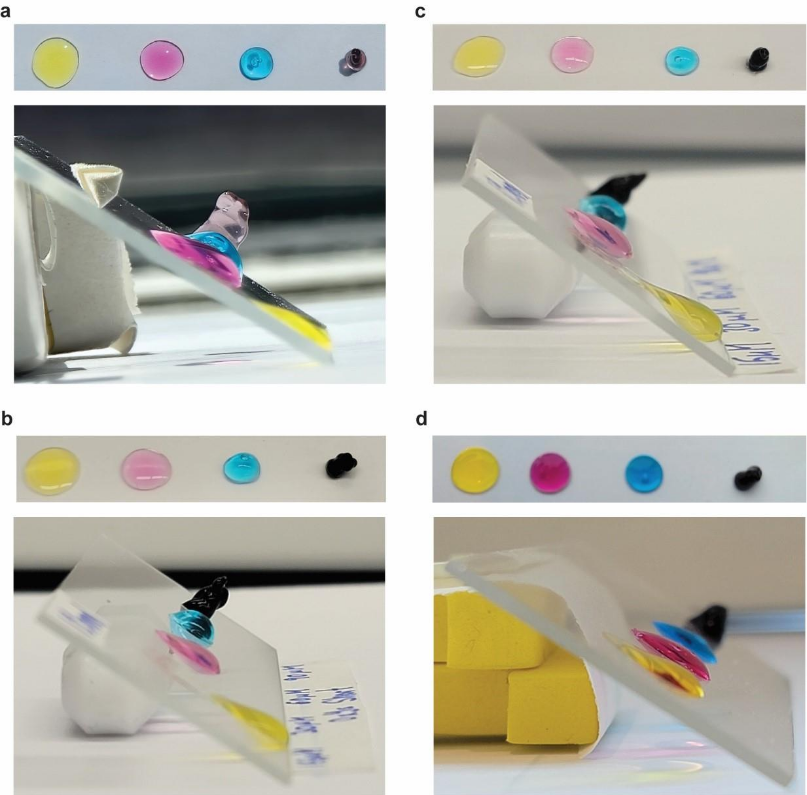


**Figure S6.** **Representative 3WJ gel array images on flat (top panel) and tilted (bottom panel) surfaces.** The 40 µL samples were made at 15 µm (yellow), 30 µm (pink), 60 µm (blue), and 90 µm (black). **a)** 3WJ-Bl. **b)** 3WJ-Sh. **c)** 3WJ-Lo. **d)** 3WJ-Lm.


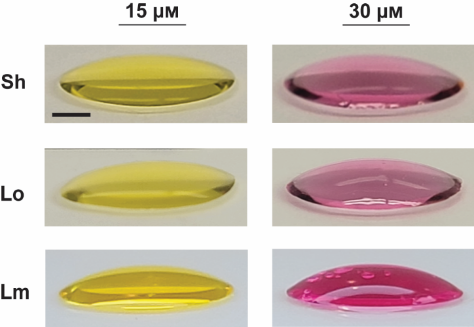


**Figure S7.** **Representative 3WJ gel array images at 15 and 30 µm.** All samples were made at 40 µL. (Scale bar: 5 mm).


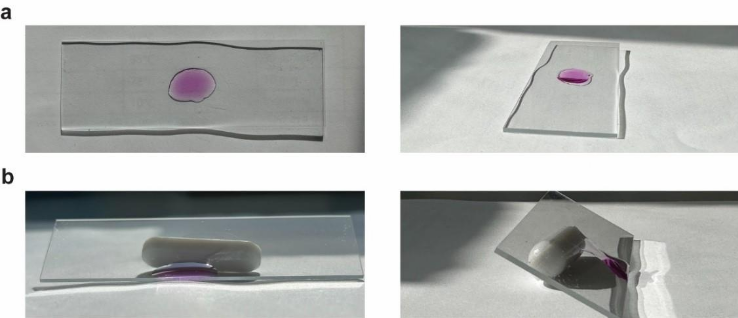


**Figure S8.** **3WJ mixture with single motif type (noncomplementary motifs) fail to form hydrogel.** Representative images of 3WJ-Lm at 60 µm motif B on **a)** flat and **b)** tilted surfaces. The two samples were made at 40 µL.


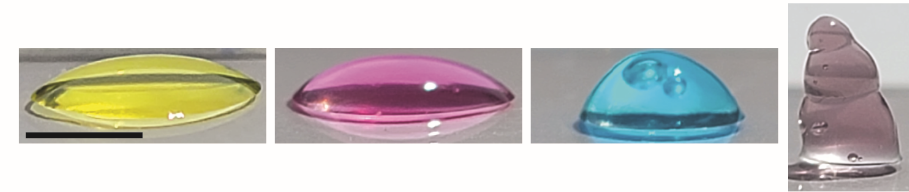
**Figure S9.** **Representative full gel array images of 3WJ-Bl hydrogel.** From left to right: 15 µm, 30 µm, 60 µm, and 90 µm (Scale bar: 5 mm). All sample were made at 40 µL.


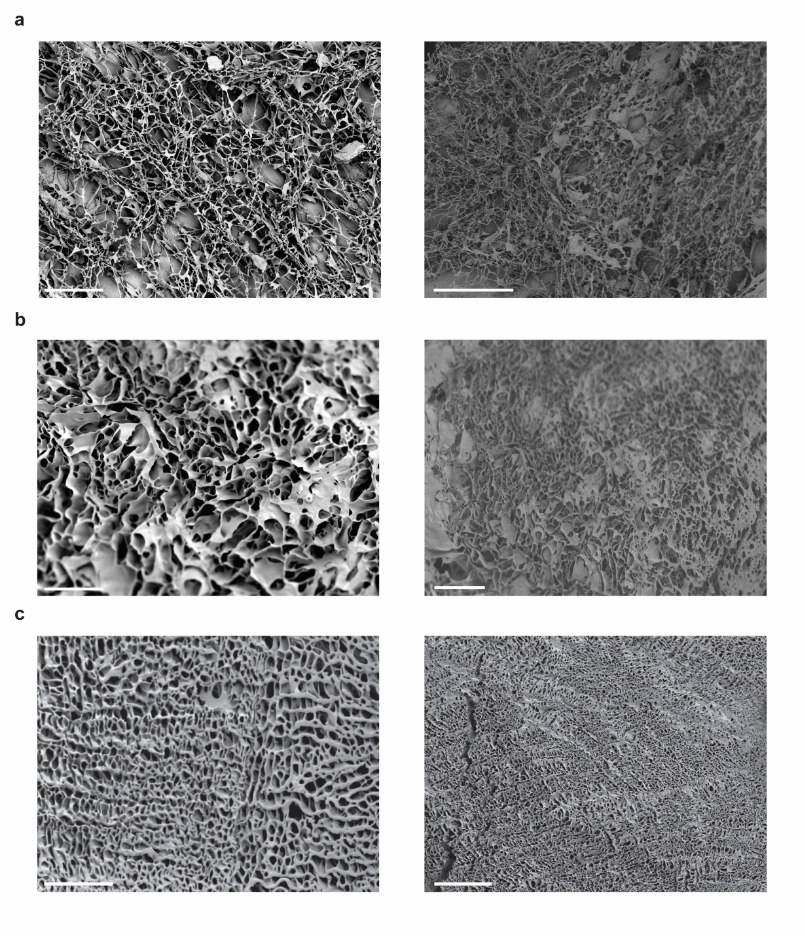


**Figure S10.** **Representative (*left*; scale bar: 50 µm) and full (*right*; scale bar: 100 µm) scanning electron microscopy (SEM) images of 3WJ-Bl hydrogel.** **a)** 30 µm, **b)** 60 µm, and **c)** 90 µm.


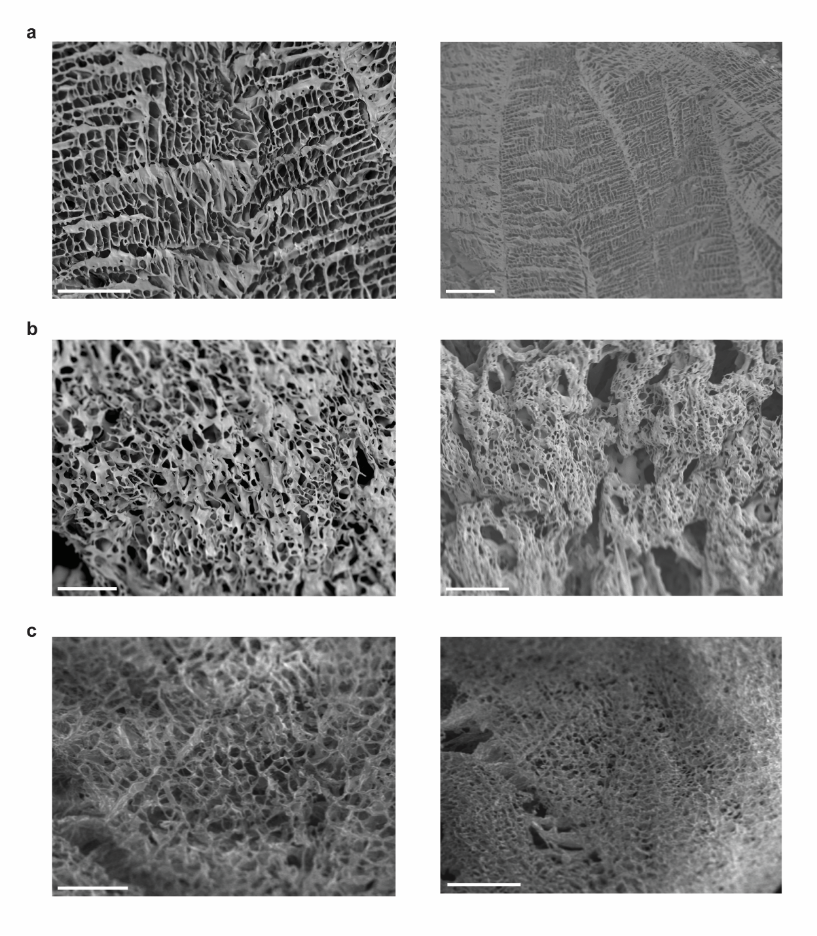


**Figure S11.** **Representative (*left*; scale bar: 50 µm) and full (*right*; scale bar: 100 µm) SEM images of 3WJ hydrogels at 30 µm. a)** Sh, **b)** Lo, and **c)** Lm linkers.


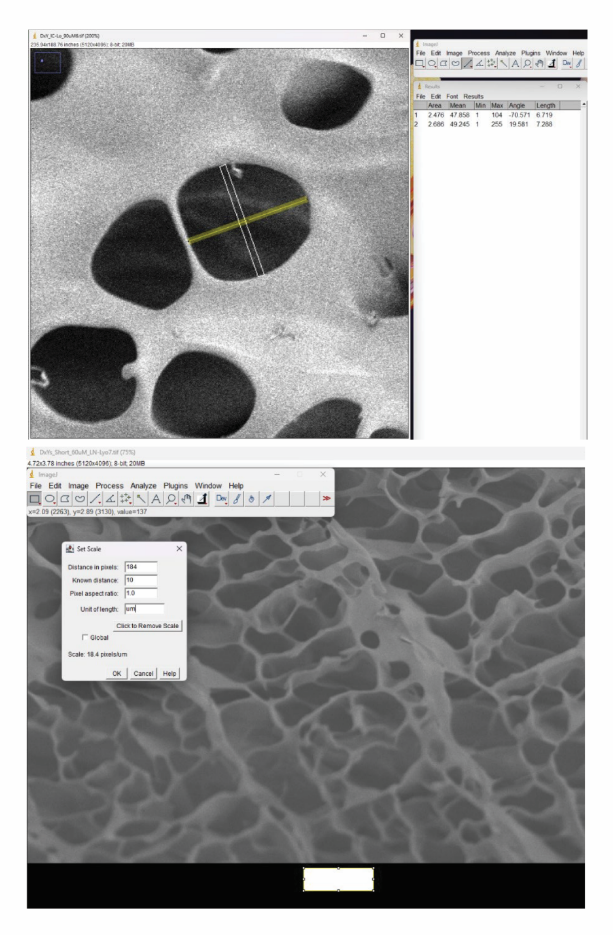


**Figure S12.** **Pore size measurement procedure used for SEM images of DX-tile hydrogels.** Protocol followed for pore size measurements using ImageJ and described in the Methods section.


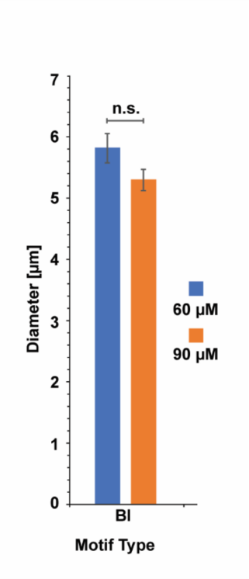


**Figure S13.** **Average pore size diameter for 3WJ-Bl hydrogels.** Error bars represent standard deviation of the mean (*n* = 50 independent samples/group) and p values were calculated from a student t-test (not significant, n.s.).


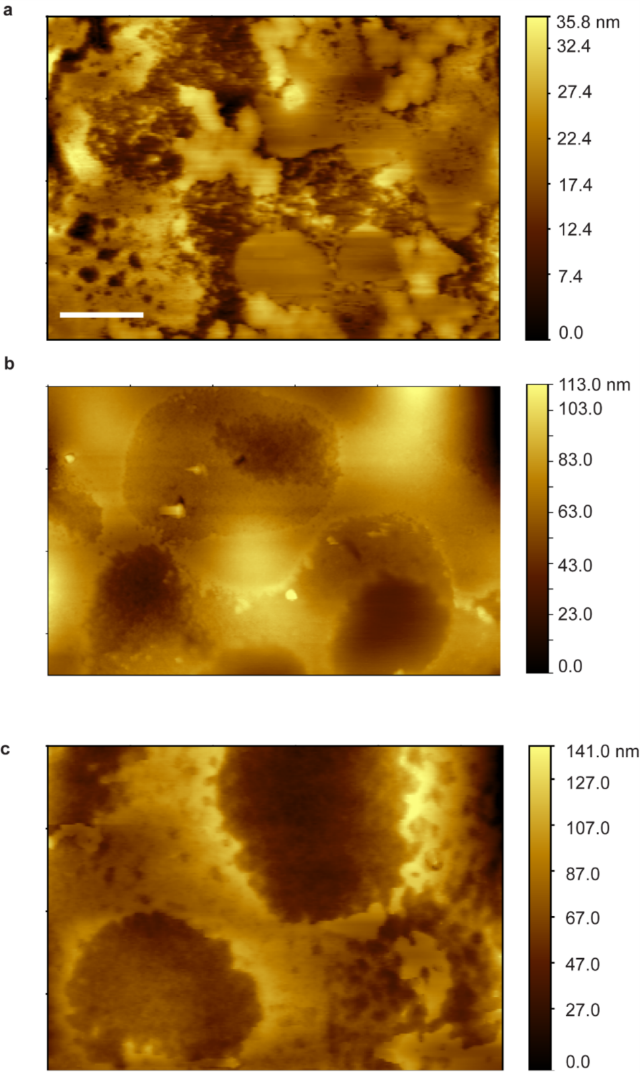


**Figure S14**. **Full-size AFM images of vacuum-dried 3WJ hydrogels. a)** Sh, **b)** Lo, and **c)** Lm (scale bar: 1 µm). All samples imaged at 60 µm.


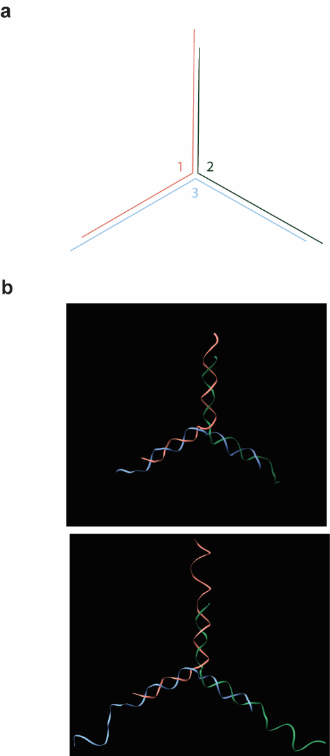
[Text Wrapping Break]

**Figure S15. Single duplex Y-motifs used in this study. a)** Numbering schematic. **b)** Three dimensional models of the Y7 (*top*) and Y21 (*bottom*) motifs.


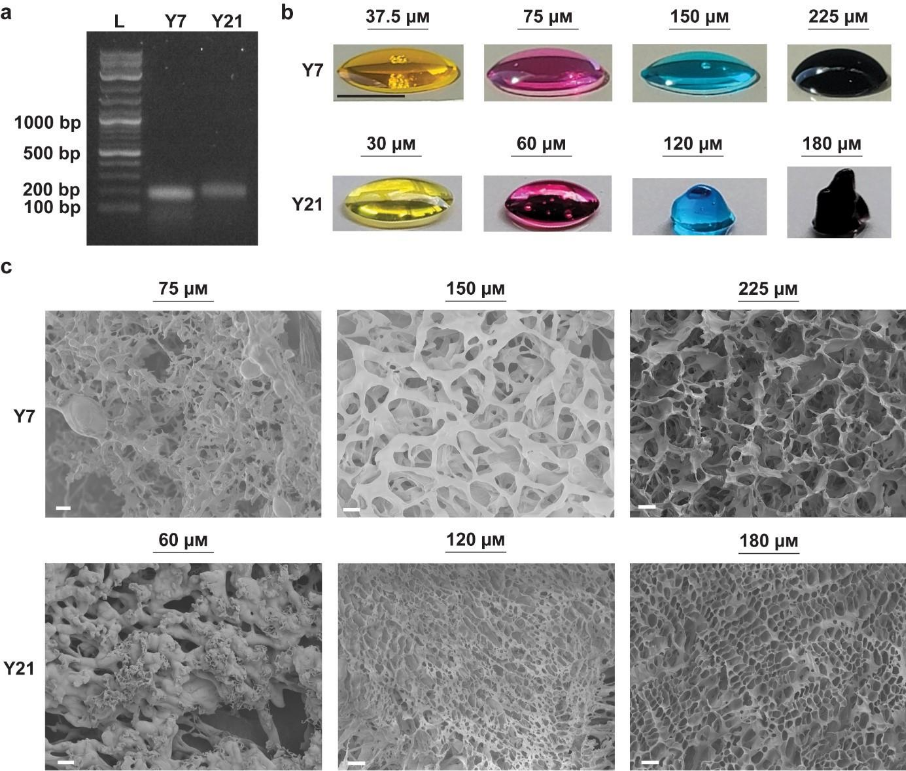


**Figure S16.** **Y motif folding and hydrogel formation. a)** Agarose gel electrophoresis confirming motif folding. **b)** Array images of hydrogels at comparable concentrations. **(**Scale bar: 5 mm). **c)** Representative SEM images verifying hydrogel formation. (Scale bars: 20 µm).


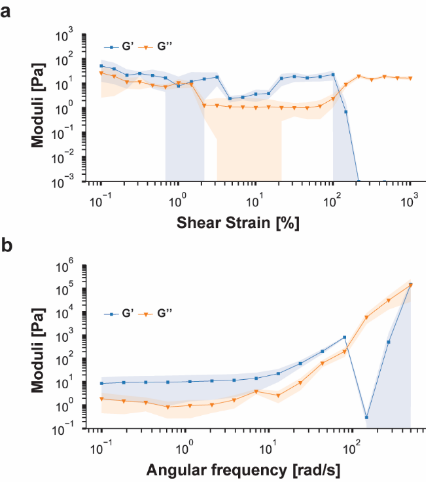


**Figure S17.** **Rheological profile of single DX-tile motif (3WJ-Sh, 60 µm)**. **a)** Amplitude sweeps. Error shadings represent standard deviation of the mean (*n* = 3 independent samples/group). **b)** Frequency sweeps. Error shadings represent standard deviation of the mean (*n* = 3 independent samples/group).


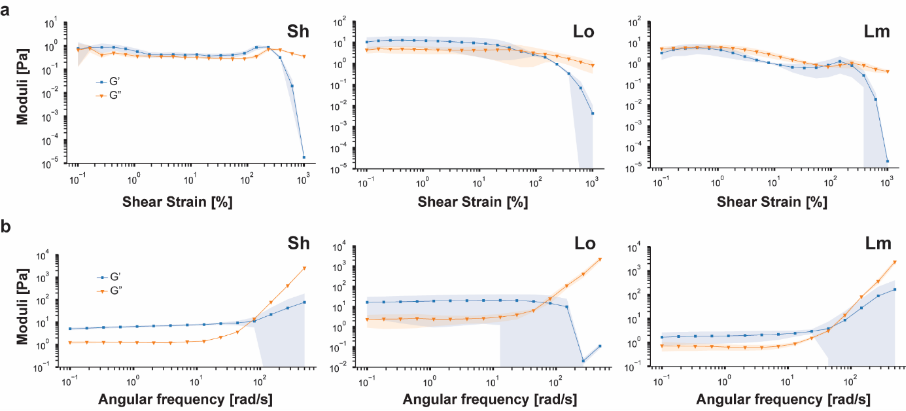


**Figure S18**. **Rheological characterization profile of the 3WJ constructs at 30 µm.** **a)** Amplitude sweeps. Error shadings represent standard deviation of the mean (*n* = 3 independent samples/group). **b)** Frequency sweeps. Error shadings represent standard deviation of the mean (*n* = 3 independent samples/group).


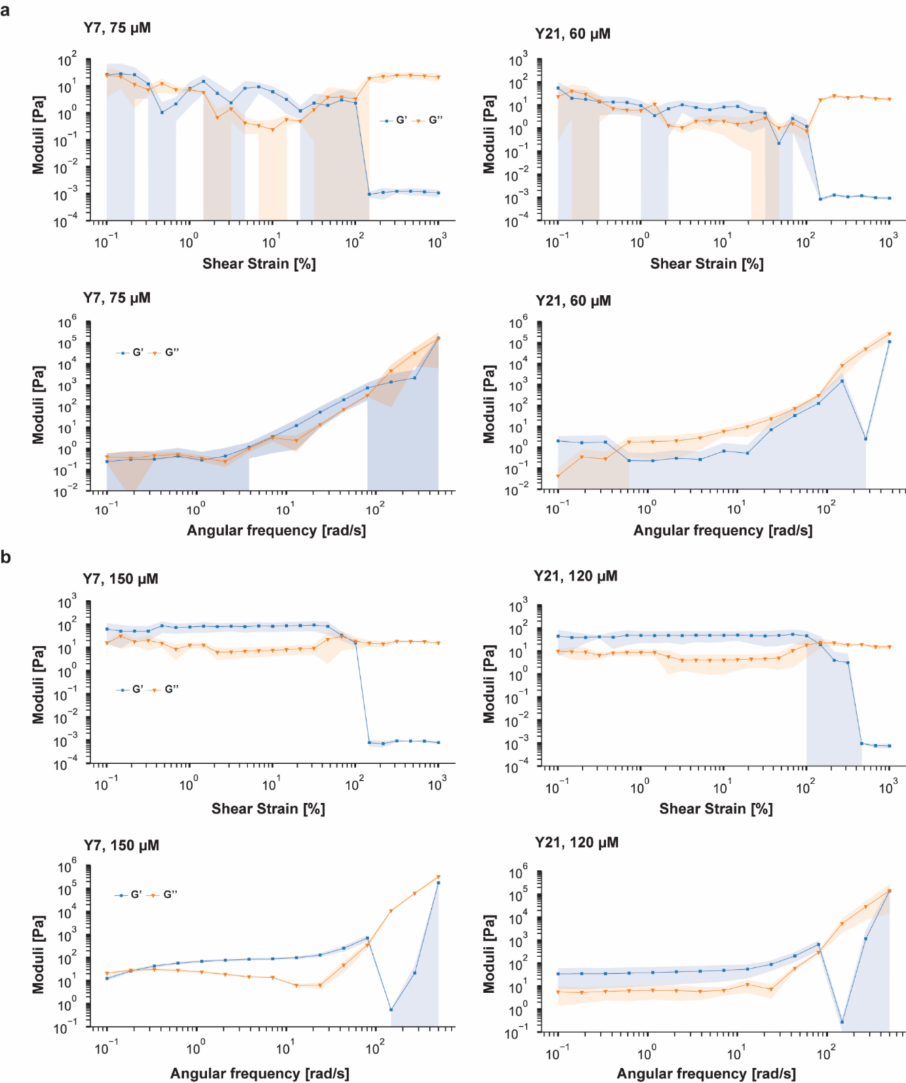


**Figure S19.** **Rheological profile of Y7 and Y21 hydrogels.** **a)** Amplitude (*top*) and frequency sweeps (*bottom*) of Y7 (*left*) and Y21 (*right*) hydrogels at 75 and 60 µm, respectively. Error shadings represent standard deviation of the mean (*n* = 3 independent samples/group). **b)** Amplitude (*top*) and frequency sweeps (*bottom*) of Y7 (*left*) and Y21 (*right*) hydrogels at 150 and 120 µm, respectively. Error shadings represent standard deviation of the mean (*n* = 3 independent samples/group).


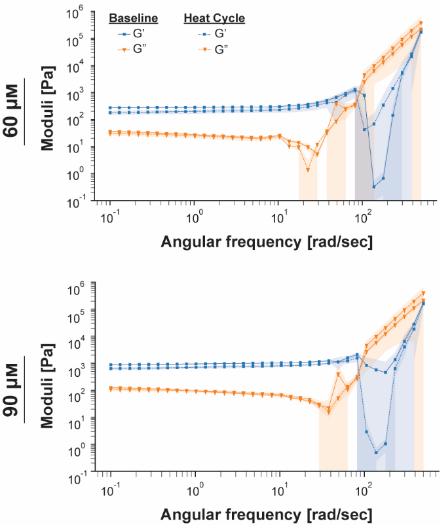


**Figure S20**. **Rheological characterization profile of the 3WJ hydrogel pre- and post- heat cycling**. Frequency sweeps of 3WJ-Sh hydrogel performed for 60 µm (top) and 90 µm (bottom). Error shadings represent standard deviation of the mean (*n* = 3 independent samples/group).


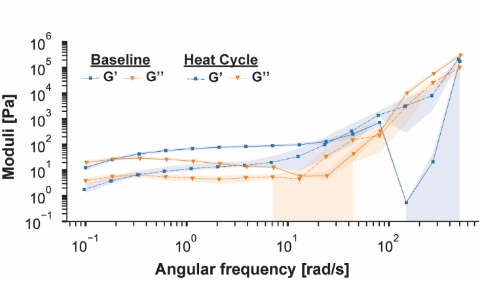


**Figure S21.** **Rheological characterization profile of the Y7 hydrogel pre- and post- heat cycling**. Frequency sweep of Y7 hydrogel (150 µm). Error shadings represent standard deviation of the mean (*n* = 3 independent samples/group).


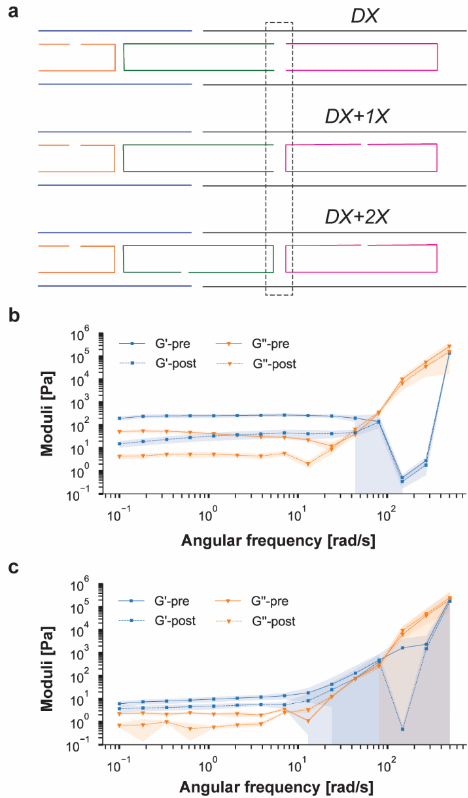


**Figure S22**. **Design schematic and rheological characterization profile of the 3WJ with the single and double crossovers**. **a)** Schematic of the original DX arm (*top*) compared to that with one additional crossover (*1X; middle*) and two additional crossovers (*2X; bottom*). **b)** Frequency sweeps of 3WJ-Sh-1X at 60 µm pre- and post-heat cycle. Error shadings represent standard deviation of the mean (*n* = 3 independent samples/group). **c)** Frequency sweeps of 3WJ-Sh-2X at 60 µm with two additional crossovers in the arms pre- and post-heat cycle. Error shadings represent standard deviation of the mean (*n* = 3 independent samples/group).


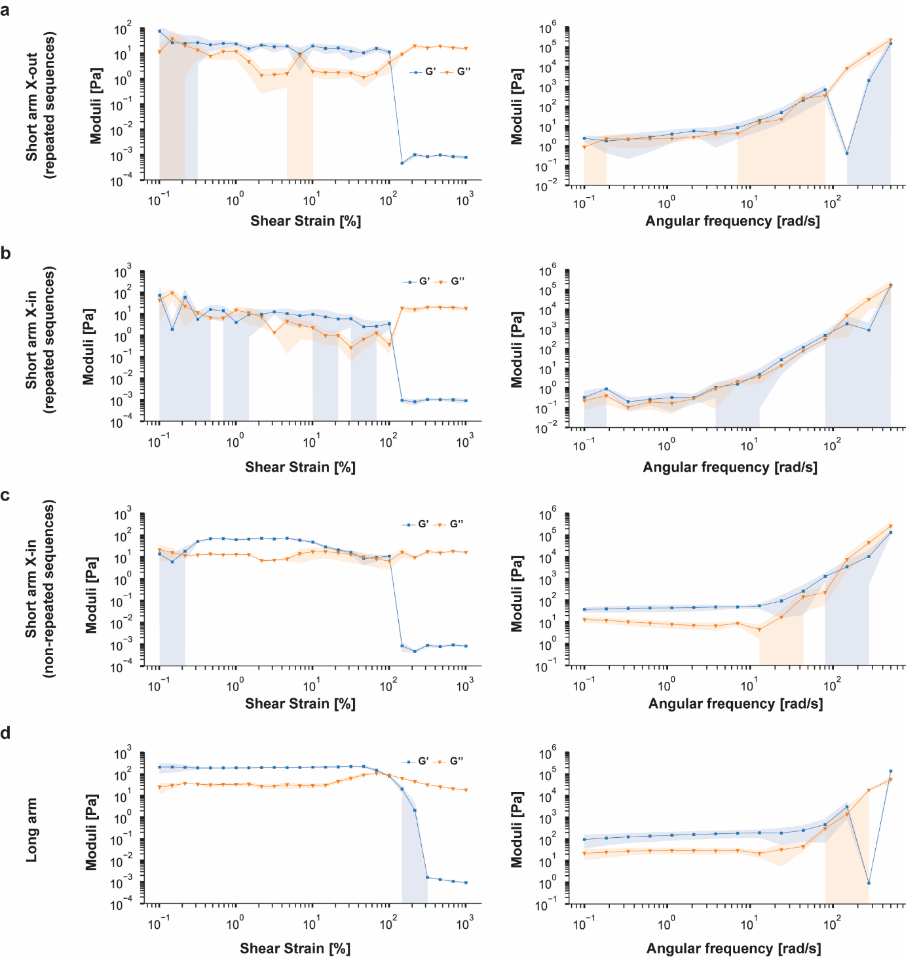


**Figure S23**. **Rheological characterization profile of the 3WJ with the short and long arms**. All samples were made with Sh linker at 60 µm. **a)** Short arm with repeating sequence outside crossover, amplitude sweep (*left*) and frequency sweep (*right*). Error shadings represent standard deviation of the mean (*n* = 3 independent samples/group). **b)** Short arm with repeating sequence inside crossover, amplitude sweep (*left*) and frequency sweep (*right*). Error shadings represent standard deviation of the mean (*n* = 3 independent samples/group). **c)** Short arm with non-repeating sequence inside crossover, amplitude sweep (*left*) and frequency sweep (*right*). Error shadings represent standard deviation of the mean (*n* = 3 independent samples/group). **d)** Long arm amplitude sweep (*left*) and frequency sweep (*right*). Error shadings represent standard deviation of the mean (*n* = 3 independent samples/group)


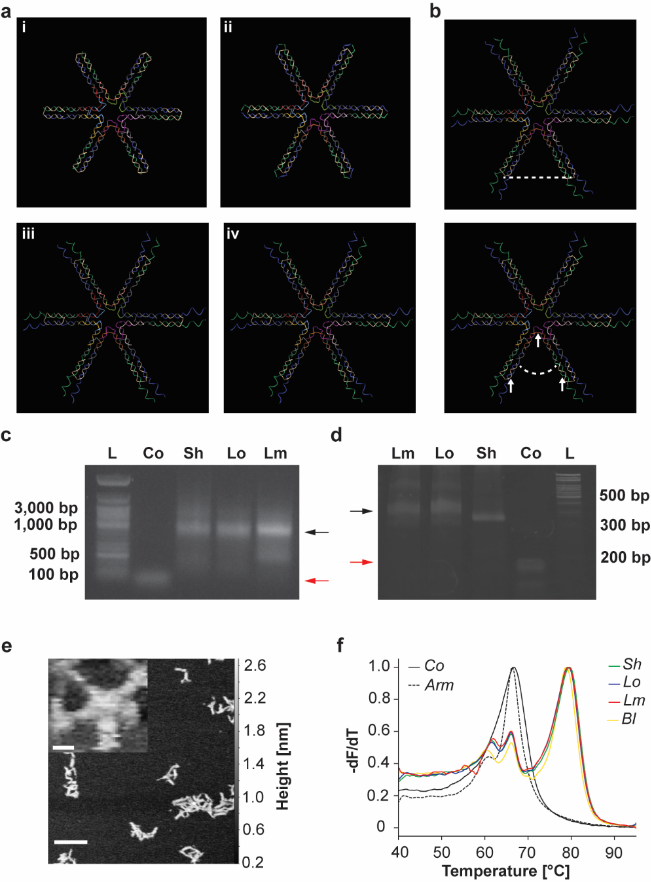


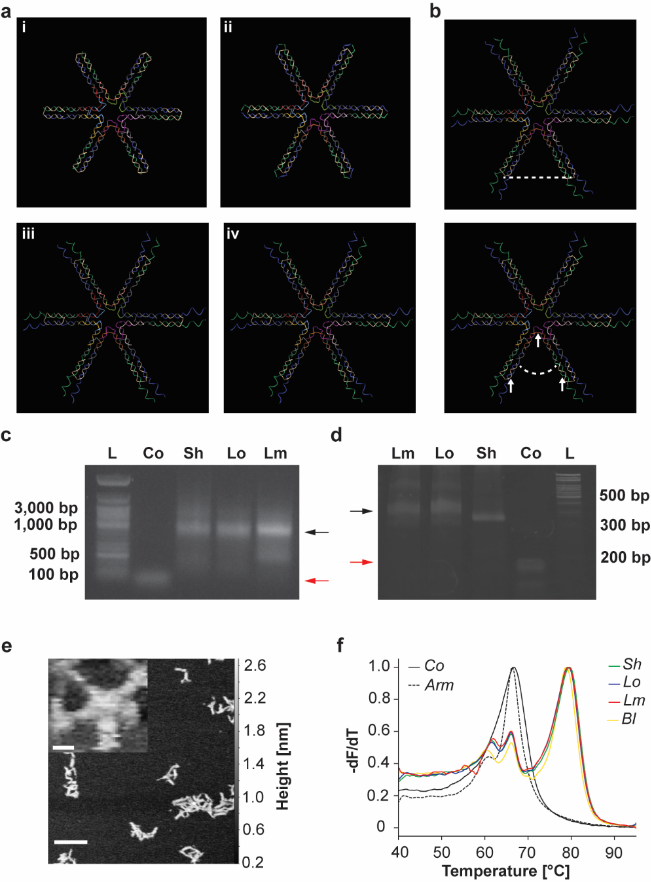


**Figure S24.** **Characterization profile of the 6WJ constructs**. **a)** 3D representation of 6WJ motifs with Bl (i), Sh (ii), Lo (iii), and Lm (iv) linkers. **b)** Estimation of the distance (*top*) and angle (*bottom*) between each arm on the 6WJ motif, shown here with the Lo linker. White arrows denote specific atoms selected for measurements. **c)** Agarose gel characterization of Core (Co) component shown along motifs folded with Sh, Lo, and Lm linker types. The red arrow indicates the folded core and the black arrow indicates the fully folded motifs. **d)** 6% PAGE gel characterization of motifs folded with Lm, Lo, and Sh linker types along with Co component. The red arrow indicates the folded core and the black arrow indicates the fully folded motifs. (L: 50 bp ladder). **e)** AFM showing folding of individual motif (full scale bar: 100 nm; inset scale bar: 10 nm). f**)** Melting curve of motif constituents (*n* = 4 independent samples/group).
